# Supplementary material for: Engineering HIV antibodies with enhanced breadth and potency of neutralization through multistate affinity maturation
Source: bioRxiv. 2025 Oct 23:2025.10.22.684031. Preprint. [Version 1] doi: 10.1101/2025.10.22.684031 (PMC12633424; doi:10.1101/2025.10.22.684031)
Supplement: 1 — Supplementary Figure 1. FACS screening strategy to enrich N49P7-FR VH dbSMS library variants with the highest affinity to six HIV gp120s. All VH variants were paired with the wild-type VL. FACS plots with sorting gates (red) and the percentage of the displayed library population selected for sorting are shown for each gp120 used at the indicated concentration. CTRL – control (unselected) population of displayed library variants not incubated with gp120 (deep sequenced for normalization to calculate mutation enrichment); AFF1 – affinity screening round 1; AFF2 – affinity screening round 2 (a sorted population was deep sequenced to calculate mutation enrichment). The experiment was performed in duplicate. Supplementary Figure 2. FACS screening strategy to enrich N49P7-FR VL dbSMS library variants with the highest affinity to six HIV gp120s. All VL variants were paired with the wild-type VH. FACS plots with sorting gates (red) and the percentage of the displayed library population selected for sorting are shown for each gp120 used at the indicated concentration. CTRL – control (unselected) population of displayed library variants not incubated with gp120 (deep sequenced for normalization to calculate mutation enrichment); AFF1 – affinity screening round 1; AFF2 – affinity screening round 2 (a sorted population was deep sequenced to calculate mutation enrichment). The experiment was performed in duplicate. Supplementary Figure 3. FACS screening strategy to enrich N49P7-FR VHv1 dbSMS library variants with the highest affinity to six HIV gp120s and enhanced thermal stability. All VHv1 variants were paired with the optimized VL (VLv3). FACS plots with sorting gates (red) and the percentage of the displayed library population selected for sorting are shown for each gp120 used at the indicated concentration. CTRL – control (unselected) population of displayed library variants not incubated with gp120 (deep sequenced for normalization to calculate mutation enrichment); AFF1 – [file NIHPP2025.10.22.684031V1-supplement-1.pdf]

Supplementary Fig. 1

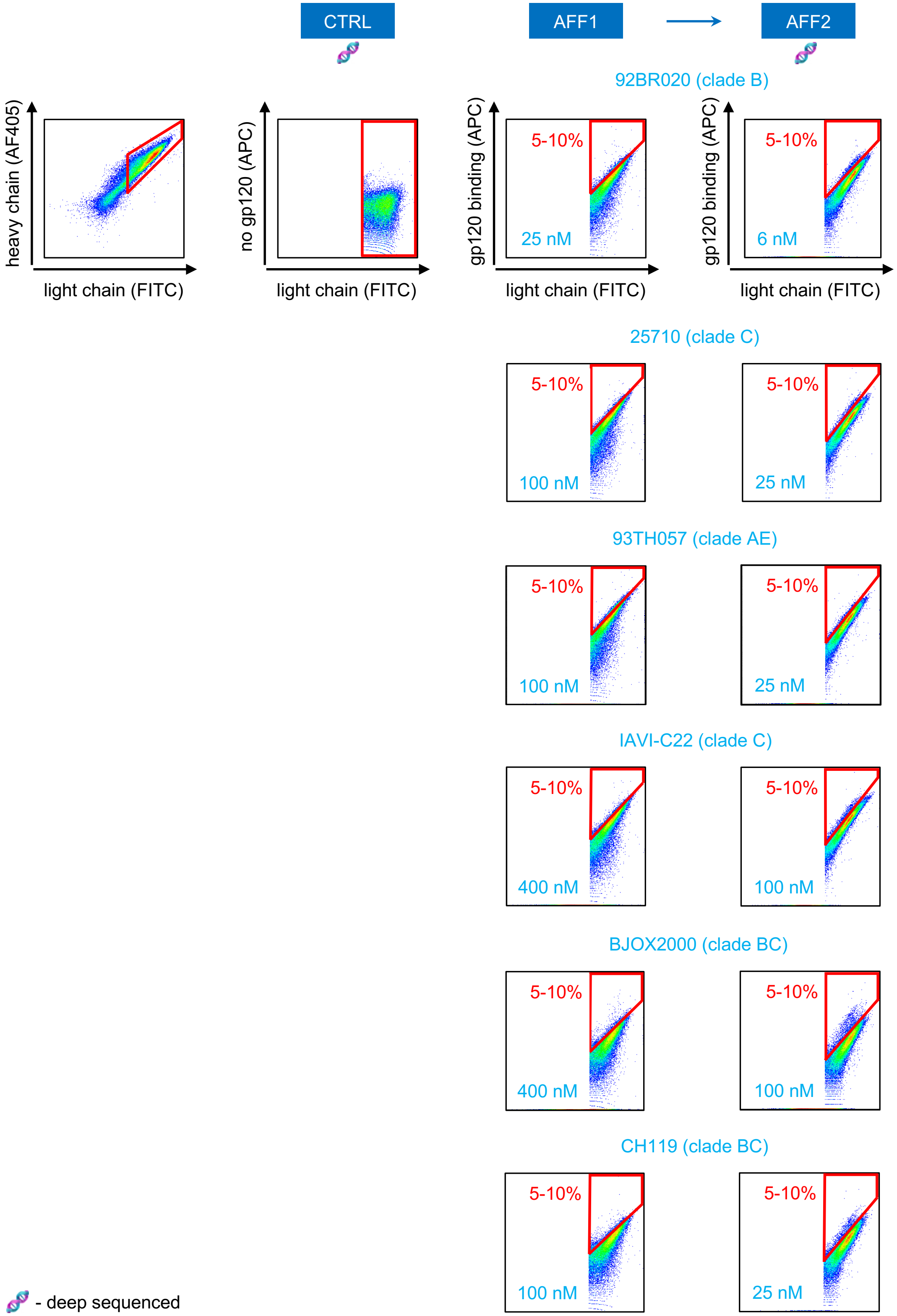

Supplementary Fig. 2

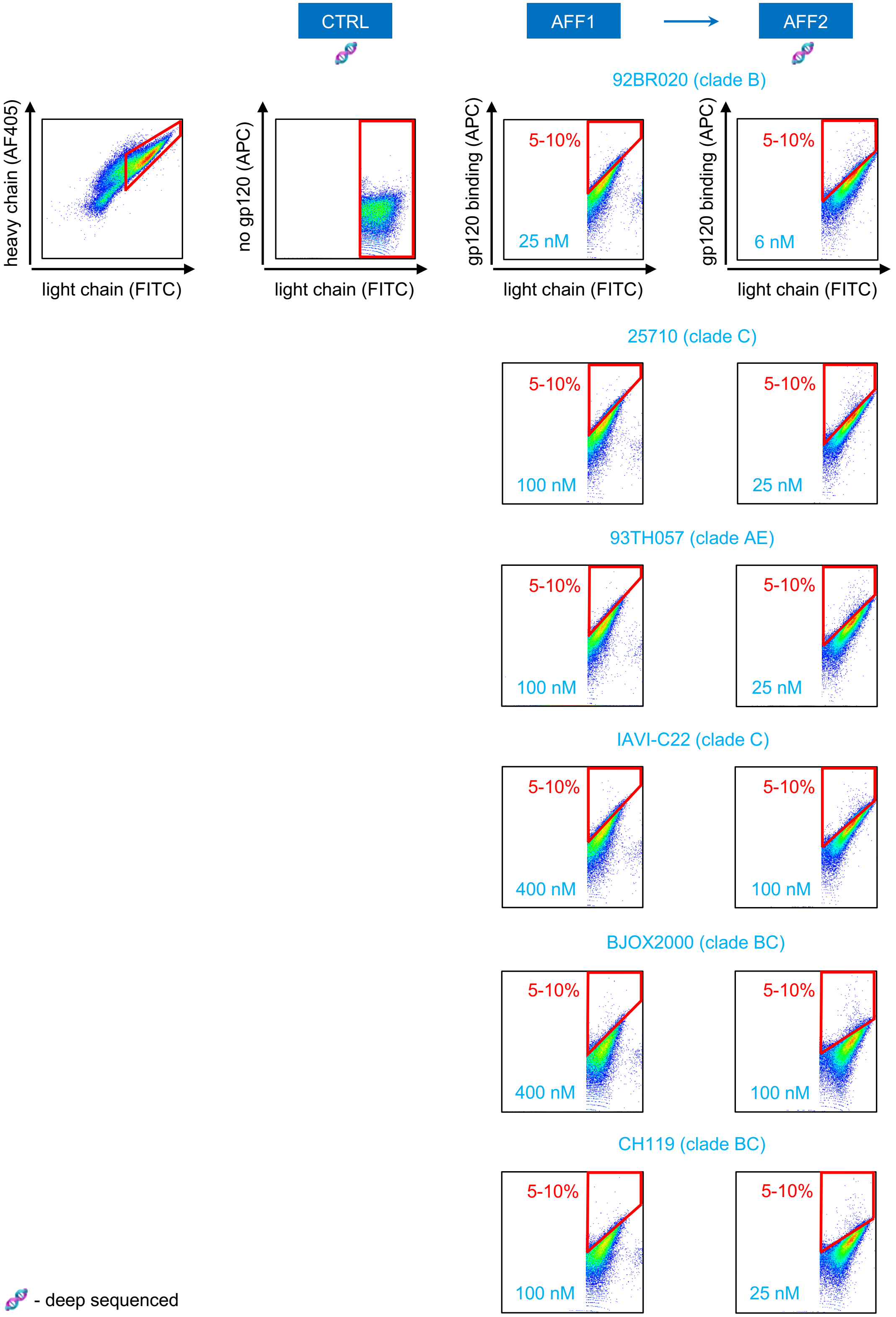

Supplementary Fig. 3

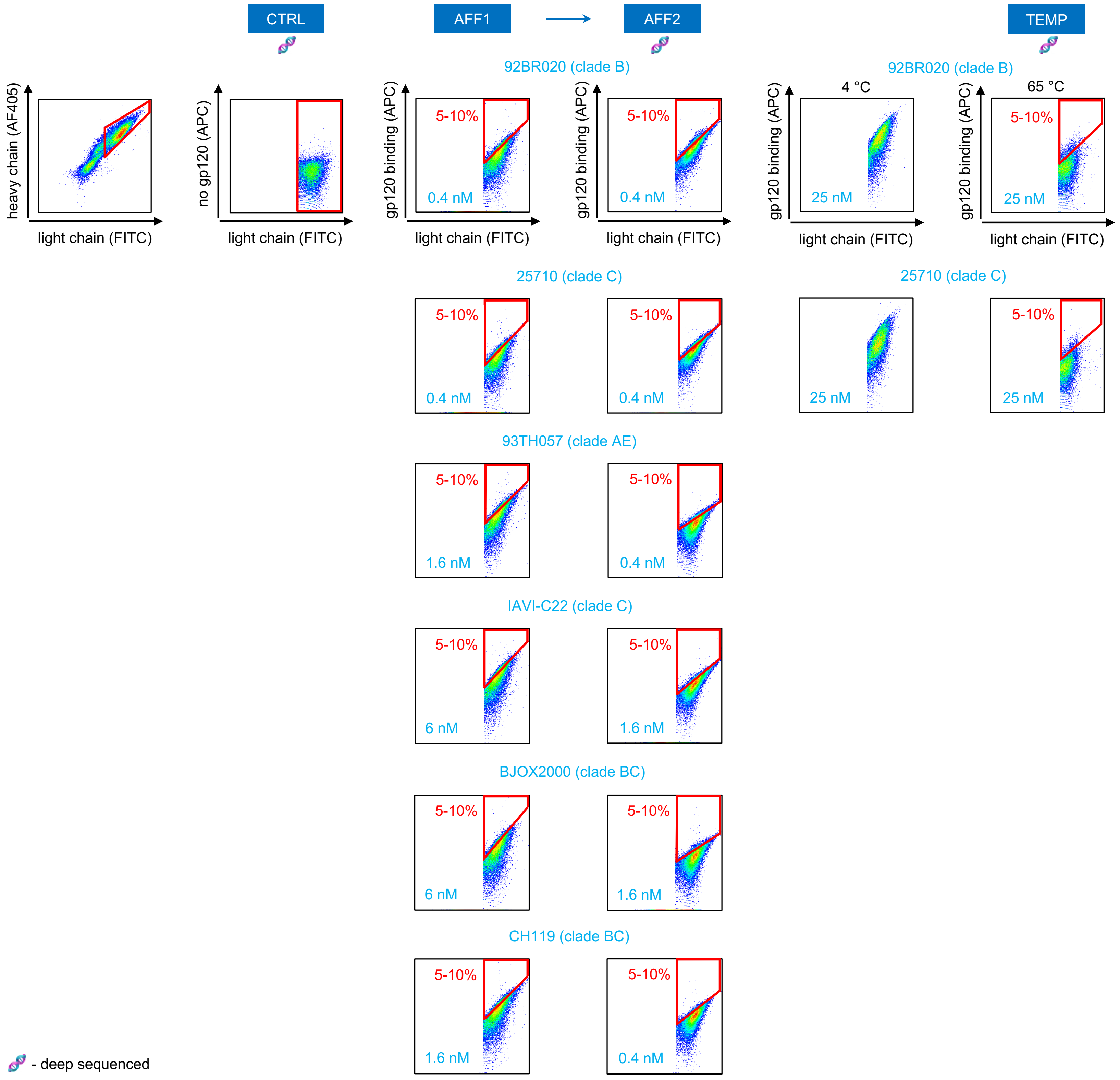

Supplementary Fig. 4

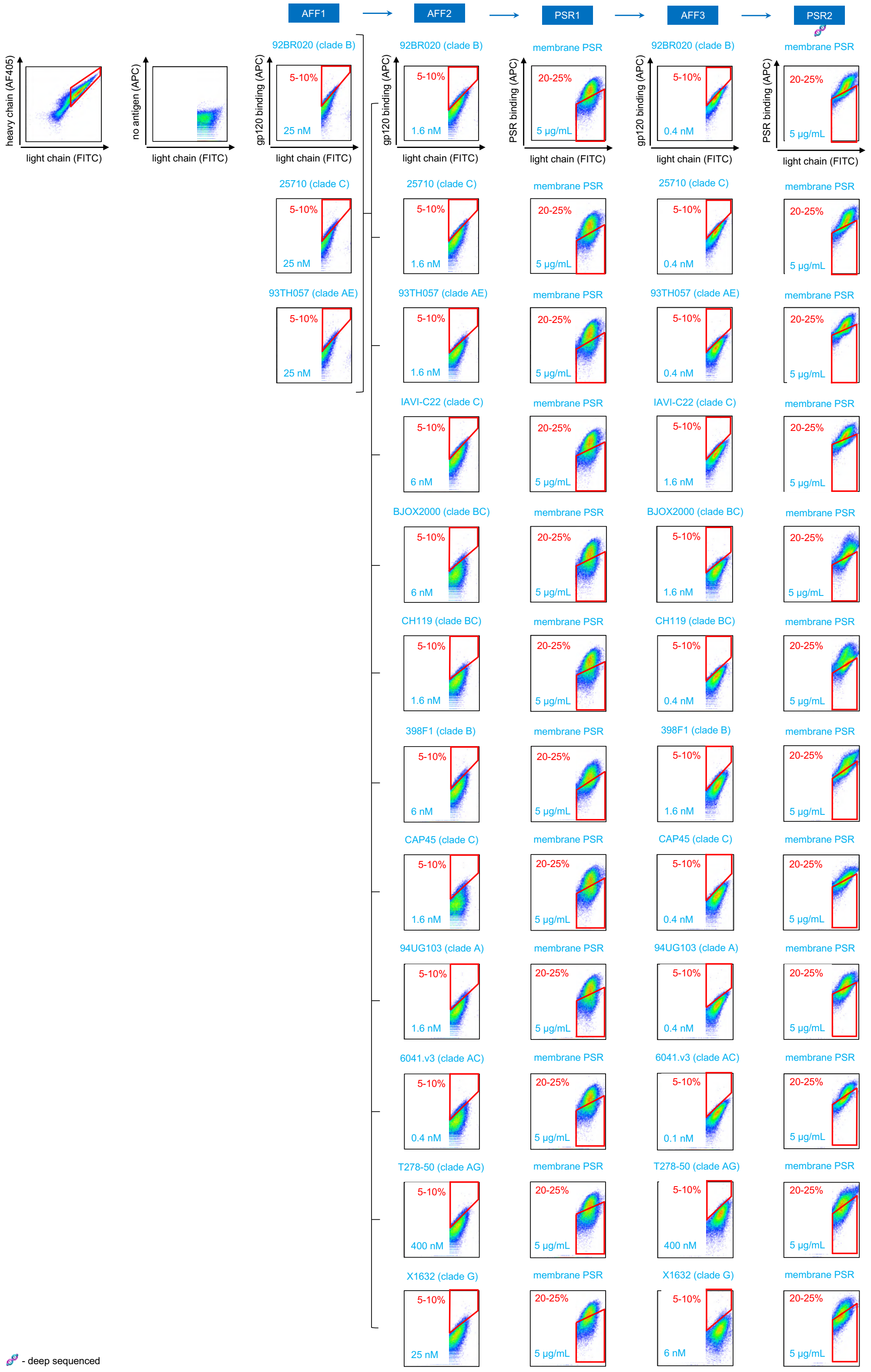

Supplementary Fig. 5

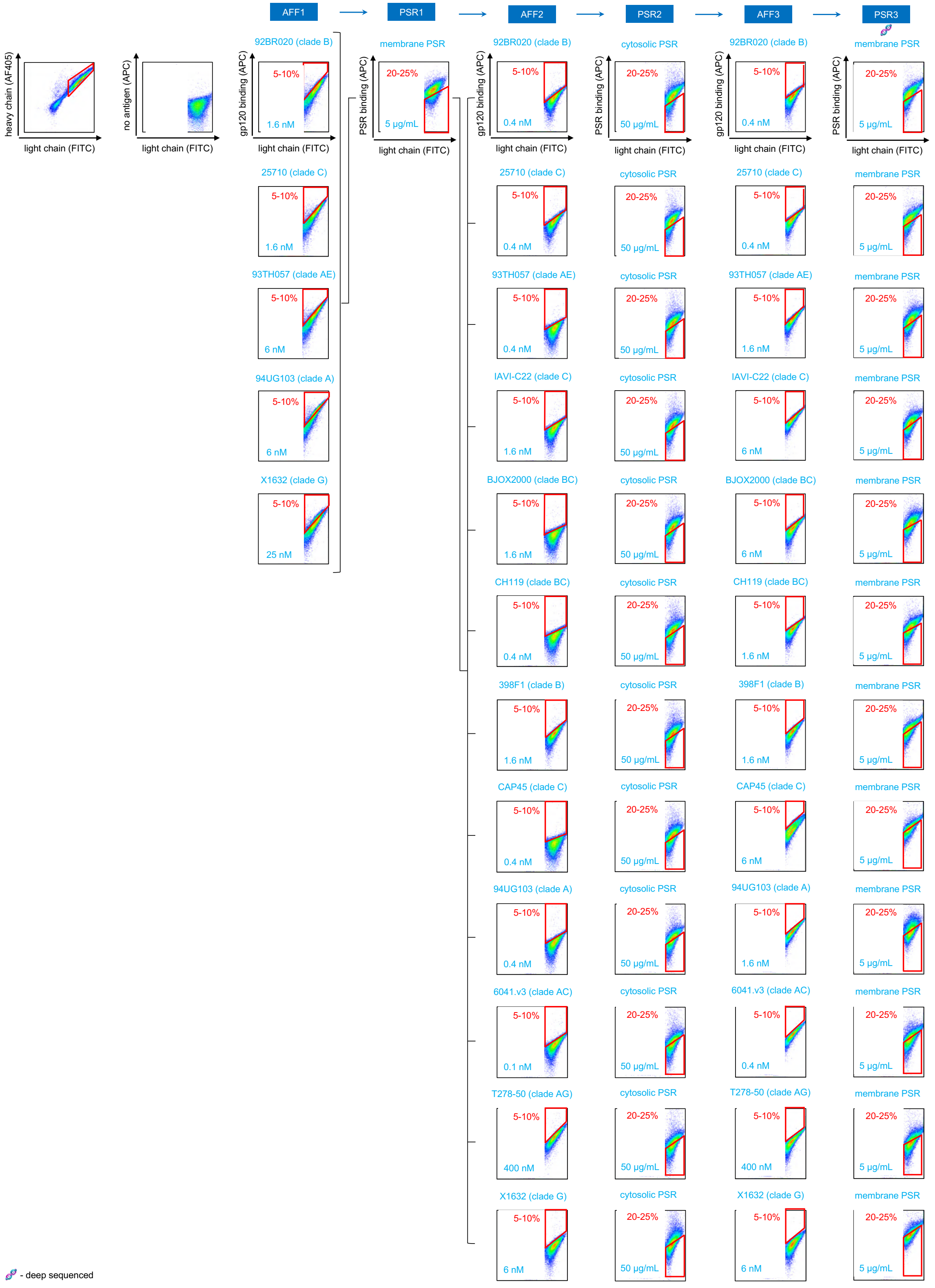

Supplementary Fig. 6

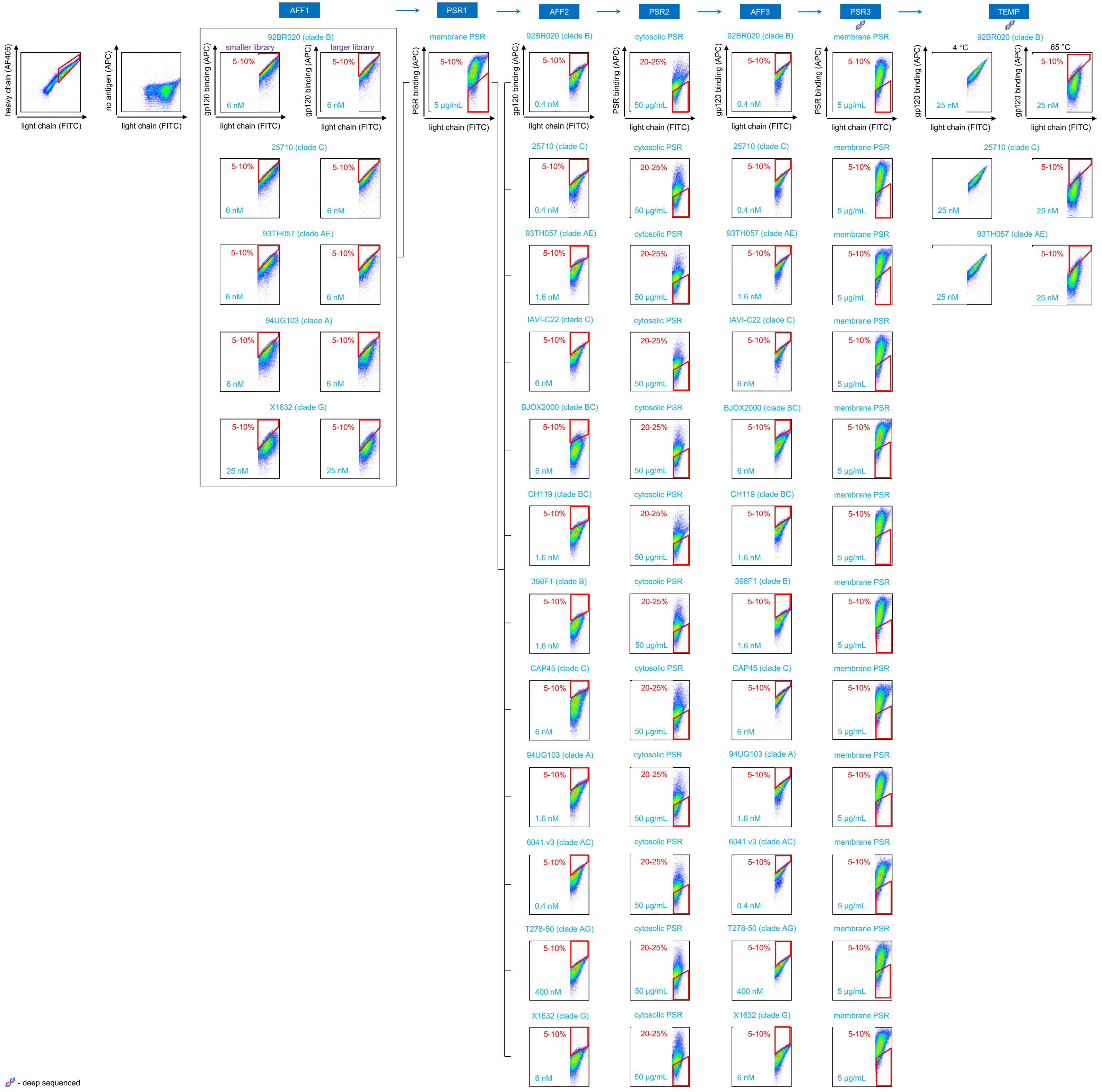

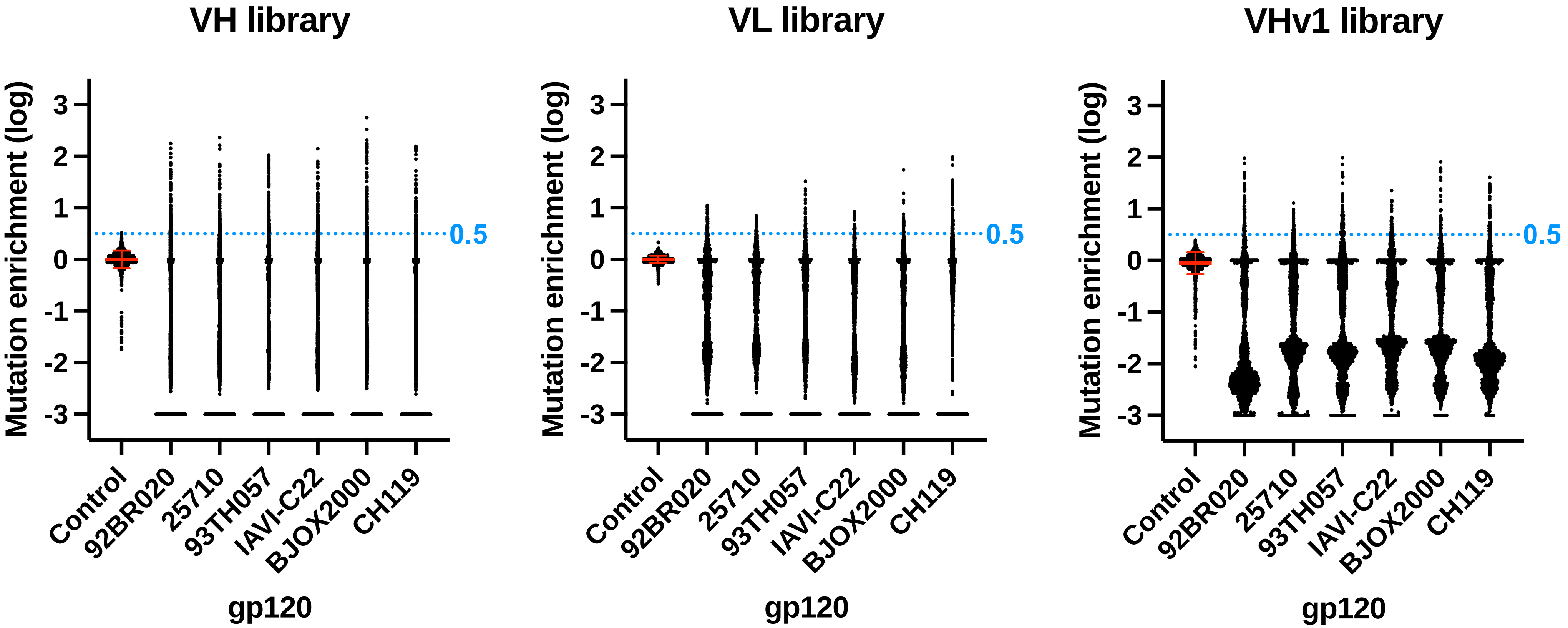
VH library
VL library
VHv1 library
Mutation enrichment (log)
3
2
1
0
-1
-2
-3
0.5
Control
92BR020
25710
93TH057
IAVI-C22
BJOX2000
CH119
gp120

Supplementary Fig. 8

92BR020 (clade B)

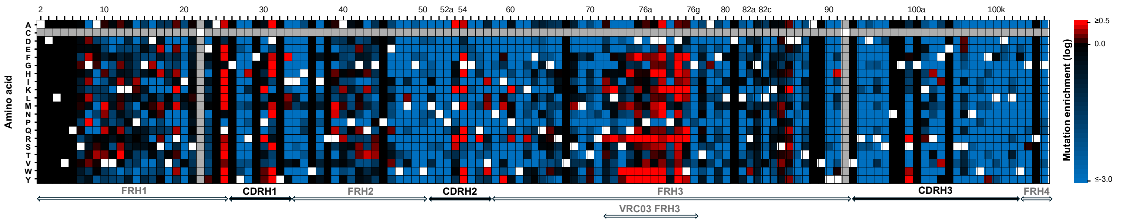

25710 (clade C)

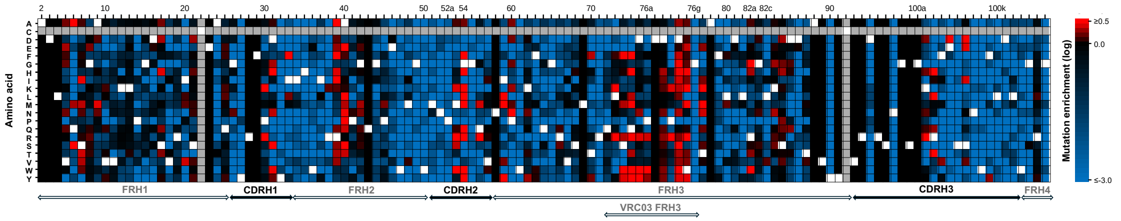

93TH057 (clade AE)

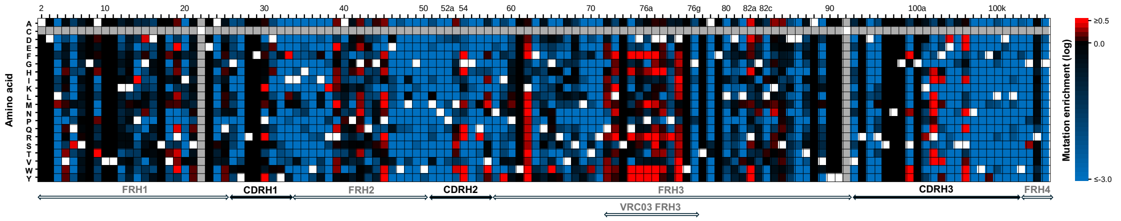

IAVI-C22 (clade C)

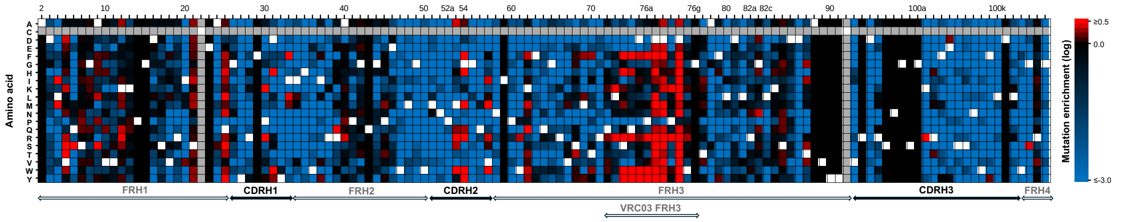

BJOX2000 (clade BC)

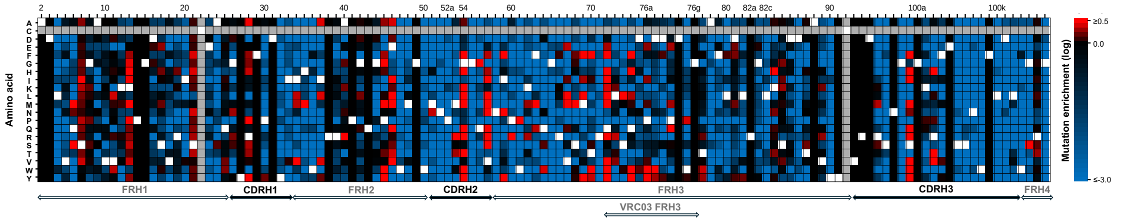

CH119 (clade BC)

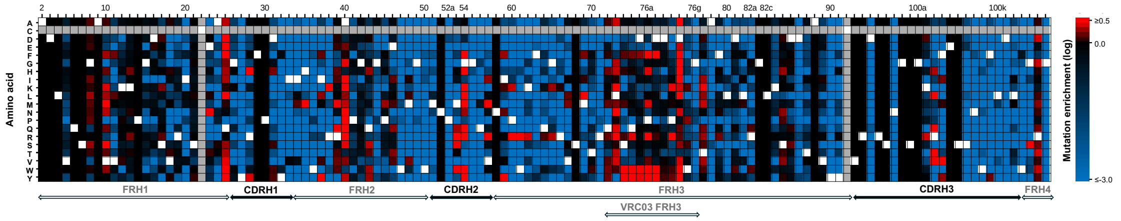

Supplementary Fig. 9

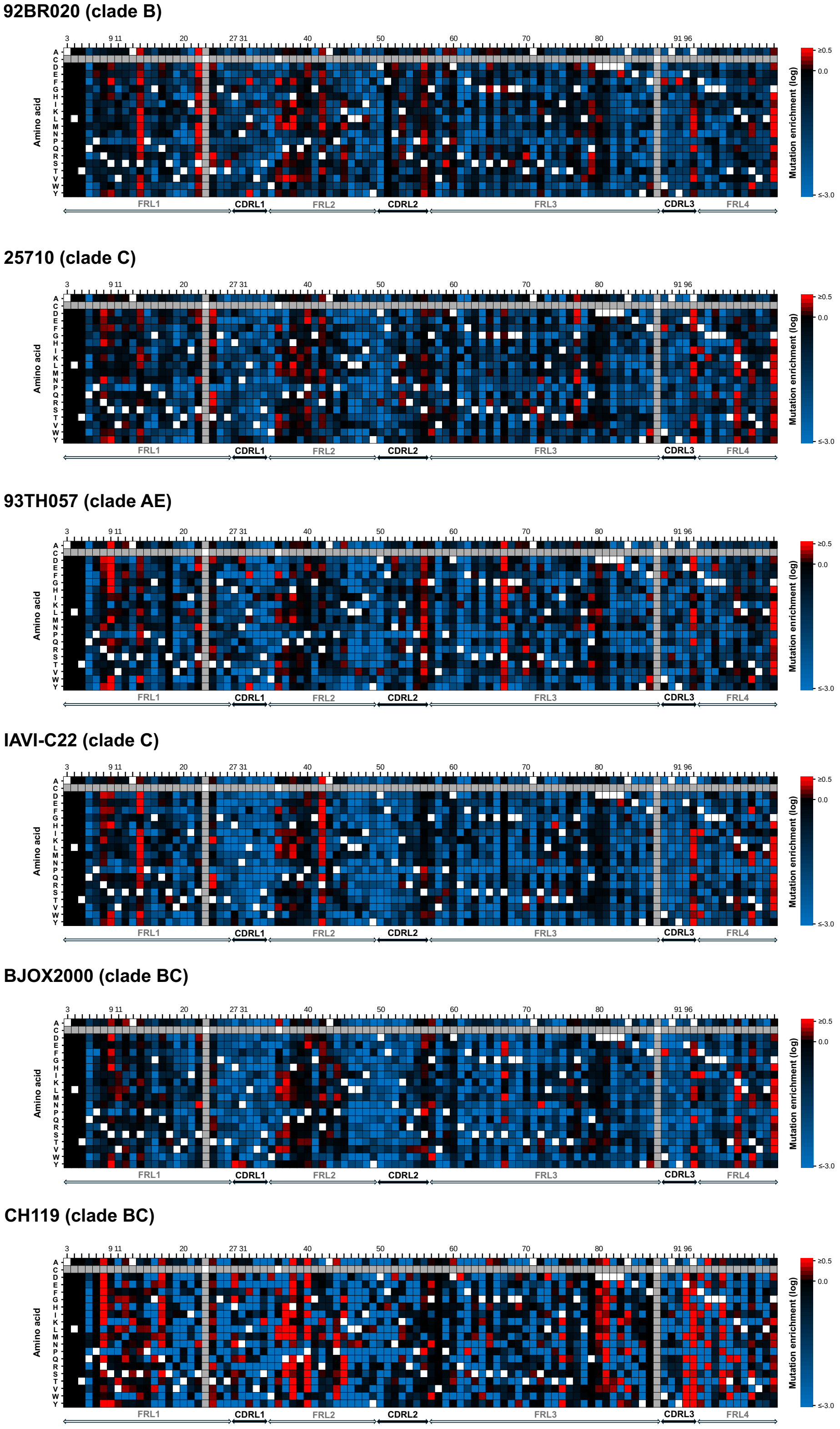

Supplementary Fig. 10

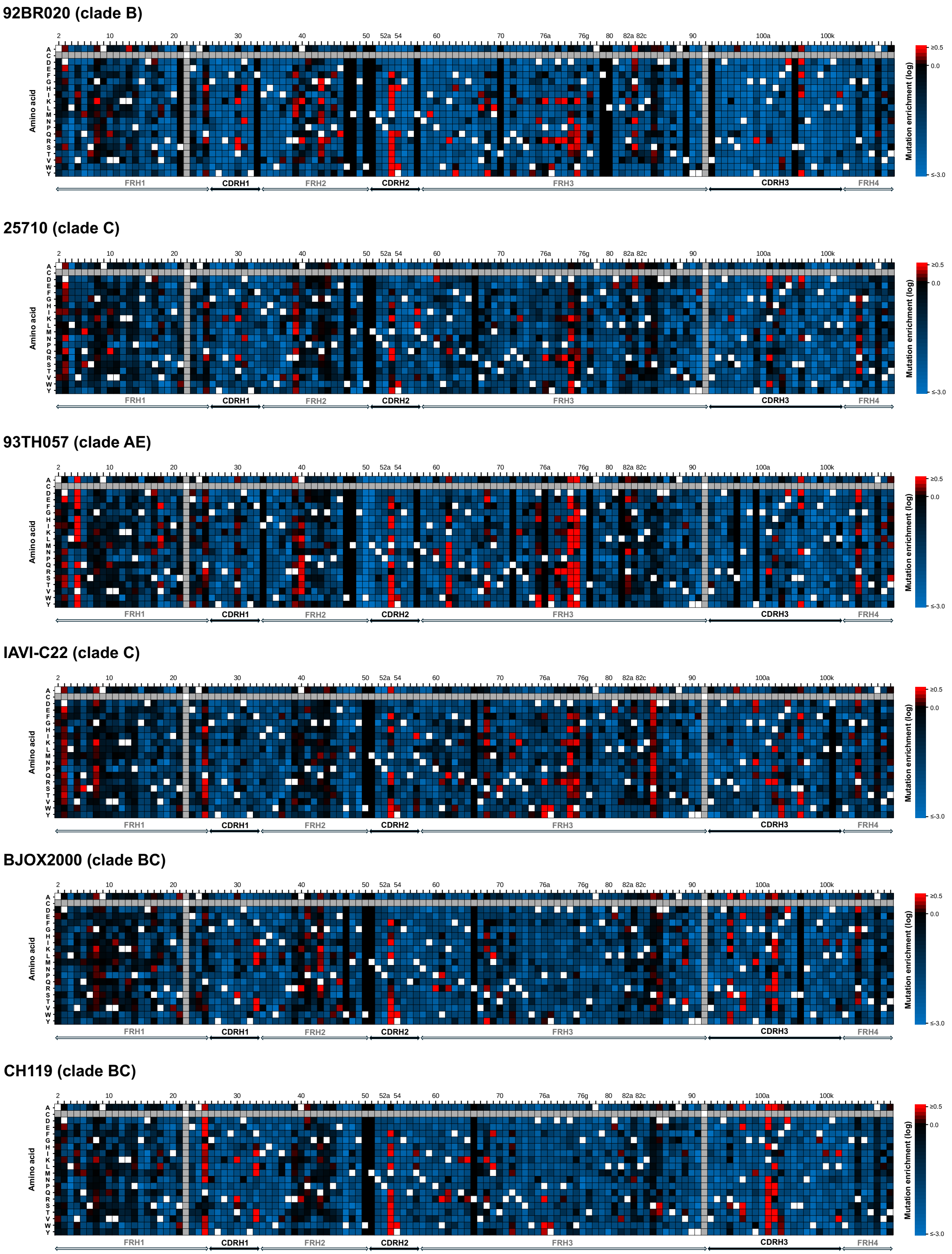

Supplementary Fig. 11

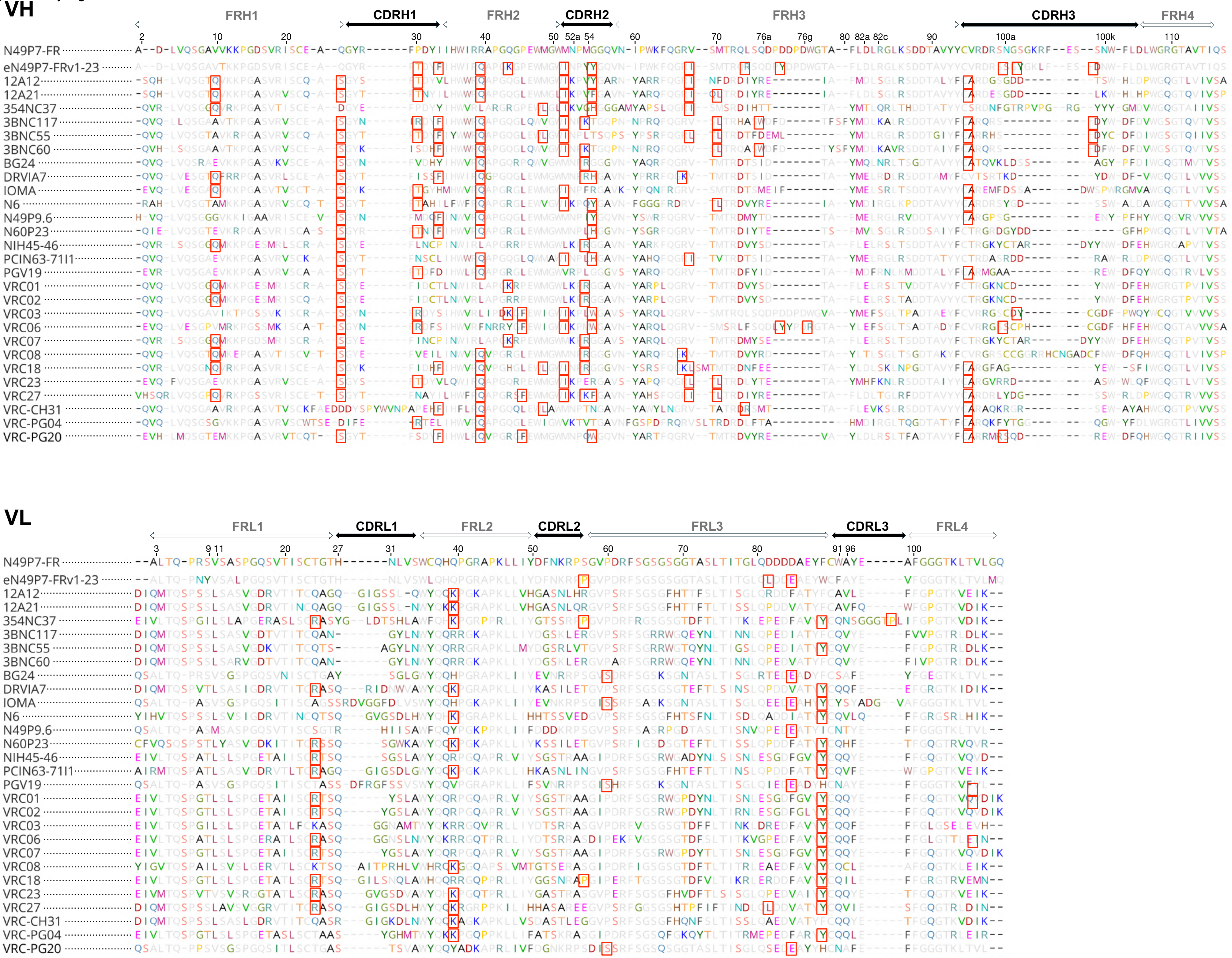

Supplementary Fig. 12

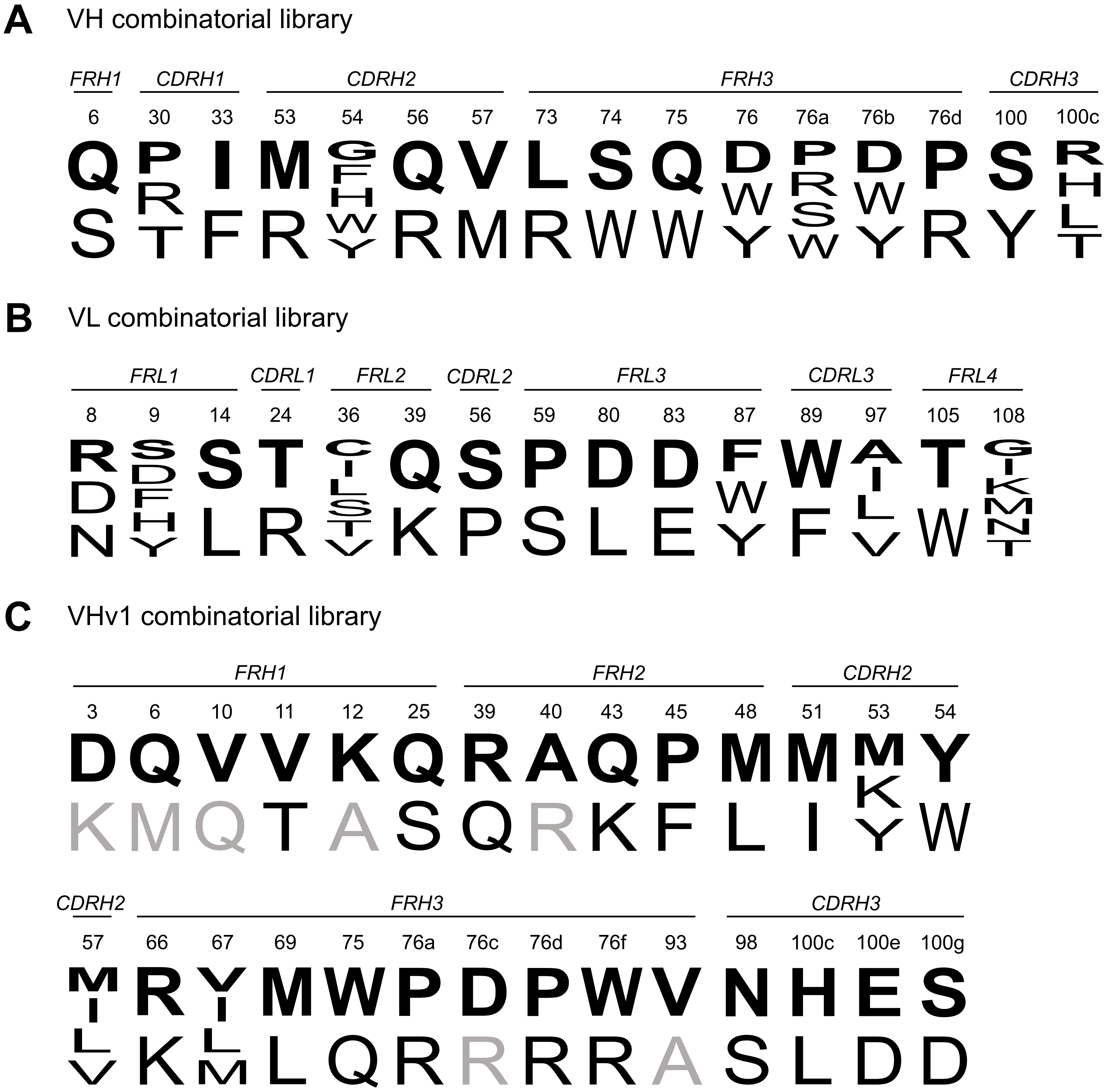

Supplementary Fig. 13

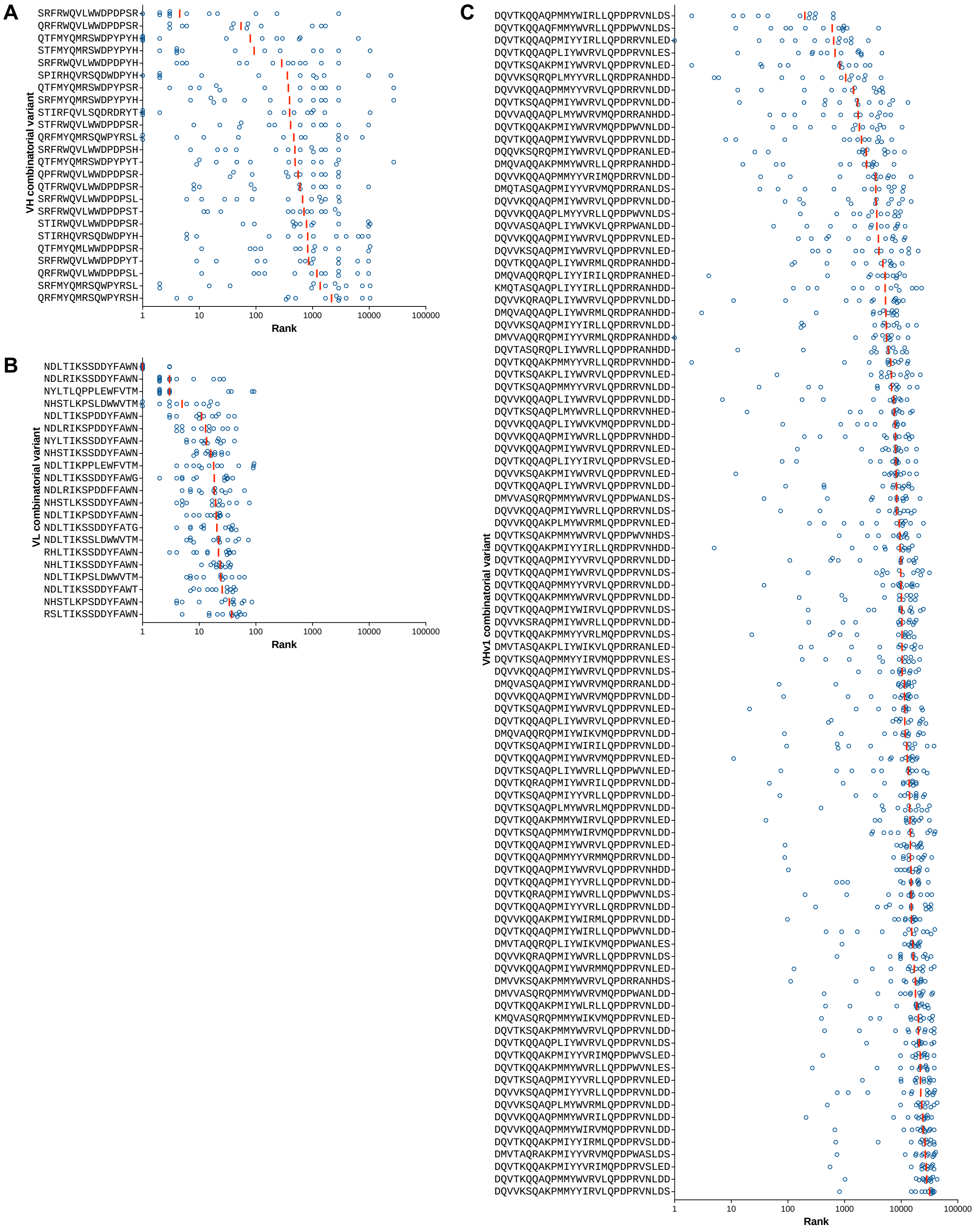

Supplementary Fig. 14

HIV pseudovirus	Clade	IC$_{80}$ [µg/mL]						
		N49P7	N49P7-FR	VHv1	VHv1+Y54G	VHv4	VHv10	VHv13
398F1	A	0.045	0.015	0.004	0.009	0.025	0.008	0.005
TRO11	B	0.042	0.007	0.004	0.007	0.012	0.005	0.004
BJOX2000	BC	3.839	0.148	0.006	0.038	0.036	0.060	0.026
Ce0217	C	0.101	0.029	0.004	0.011	0.021	0.016	0.008
Ce1176	C	0.251	0.280	0.013	0.049	0.075	0.101	0.028
CH119	BC	0.088	0.125	0.007	0.020	0.024	0.041	0.012
X1632	G	>50	>50	0.004	0.046	22.130	0.012	0.005
X2278	B	0.024	0.004	0.004	0.008	0.011	0.007	0.004
CNE8	AE	0.080	0.032	0.004	0.010	0.028	0.010	0.005
CNE55	AE	0.020	0.012	0.004	0.008	0.016	0.008	0.005
25710	C	0.128	0.060	0.005	0.016	0.021	0.029	0.010
92BR020	B	0.091	0.012	0.004	0.012	0.020	0.013	0.007
94UG103	A	0.181	0.018	0.011	0.038	0.190	0.015	0.011
JR-CSF	B	0.044	0.013	0.005	0.012	0.020	0.012	0.006

IC$_{80}$ >50 → <0.001

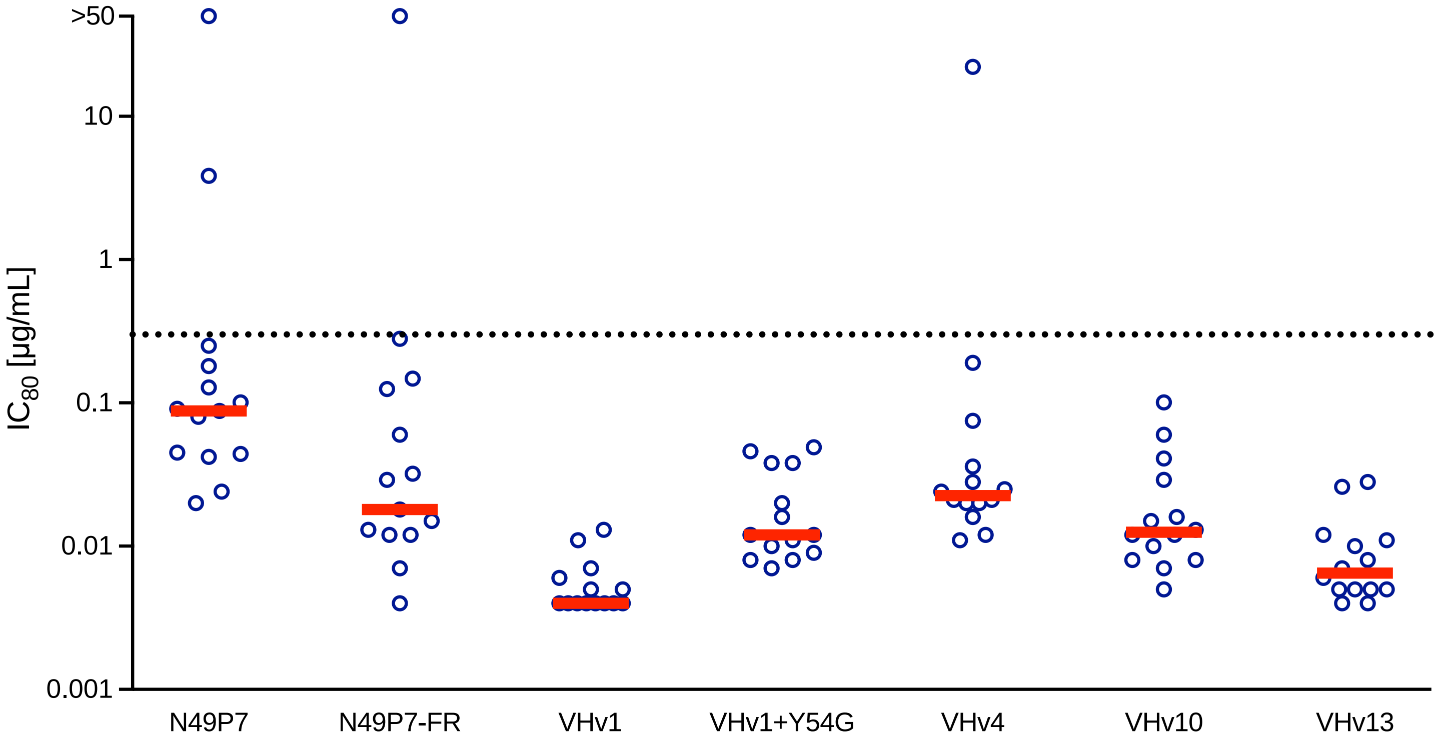

	N49P7	N49P7-FR	VHv1	VHv1+Y54G	VHv4	VHv10	VHv13
Median IC$_{80}$ [µg/mL]	0.088	0.018	0.004	0.012	0.023	0.013	0.007
GeoMean IC$_{80}$ [µg/mL]	0.096	0.027	0.005	0.016	0.043	0.016	0.008
Breadth (IC$_{80}$<0.3) [%]	86	93	100	100	93	100	100

Supplementary Fig. 15

HIV pseudovirus	Clade	IC_{80} [µg/mL]				
		N49P7-FR	VHv10/VL-WT	VHv10/VLv3	VHv10/VLv9	VHv10/VLv20
6535.3	B	0.101	0.052	0.035	0.059	0.102
SC422661.8	B	0.018	0.009	0.008	0.008	0.010
PVO.4	B	0.033	0.024	0.026	0.018	0.024
RHPA4259.7	B	0.016	0.007	0.007	0.008	0.008
REJO4541.67	B	0.025	0.018	0.014	0.014	0.017
TRJO4551.58	B	0.039	0.018	0.013	0.016	0.011
WITO4160.33	B	0.021	0.008	0.009	0.008	0.008
CAAN5342.A2	B	1.604	1.129	0.476	0.328	0.440
WEAU_d15_410_5017	B	0.013	0.009	0.008	0.007	0.009
1006_11_C3_1601	B	0.040	0.026	0.023	0.019	0.021
1054_07_TC4_1499	B	0.962	0.569	0.438	0.317	0.475
1056_10_TA11_1826	B	0.092	0.051	0.052	0.045	0.058
1012_11_TC21_3257	B	0.016	0.012	0.010	0.010	0.012
6240_08_TA5_4622	B	0.163	0.158	0.113	0.115	0.123
6244_13_B5_4576	B	0.060	0.033	0.017	0.022	0.030
62357_14_D3_4589	B	0.116	0.083	0.056	0.052	0.065
Du156.12	C	0.010	0.006	0.007	0.007	0.007
Du172.17	C	0.146	0.036	0.031	0.031	0.034
Du422.1	C	0.179	0.596	1.280	3.153	3.607
ZM197M.PB7	C	0.321	0.070	0.058	0.052	0.054
ZM214M.PL15	C	0.169	0.112	0.099	0.092	0.108
ZM249M.PL1	C	0.013	0.007	0.008	0.006	0.009
ZM53M.PB12	C	1.157	0.188	0.170	0.150	0.189
ZM109F.PB4	C	0.009	0.001	0.002	0.003	0.004
CAP45.2.00.G3	C	0.744	0.376	0.108	0.780	0.217
CAP210.2.00.E8	C	8.570	3.233	2.428	2.272	7.689
HIV-001428-2.42	C	0.006	0.004	0.004	0.004	0.004
HIV-16055-2.3	C	0.026	0.009	0.007	0.008	0.009
Ce1176_A3	C	0.280	0.101	0.051	0.044	0.068
Ce2010_F5	C	0.043	0.017	0.014	0.013	0.013
Ce703010054_2A2	C	0.143	0.032	0.025	0.023	0.026
246F C1G	C	0.033	0.010	0.009	0.011	0.014
249M B10	C	0.011	0.007	0.008	0.008	0.008
ZM247v1(Rev-)	C	0.032	0.016	0.013	0.012	0.015
Ce704809221_1B3	C	0.100	0.031	0.028	0.019	0.026
BJOX2000	BC	0.148	0.060	0.029	0.022	0.026
CH119	BC	0.125	0.041	0.020	0.019	0.031
CNE19	BC	0.026	0.008	0.008	0.007	0.008
CNE21	BC	0.161	0.040	0.031	0.040	0.044
CNE30	BC	0.156	0.073	0.055	0.055	0.075
CNE52	BC	0.005	0.004	0.004	0.004	0.004
CNE53	BC	0.025	0.009	0.008	0.011	0.010
CNE58	BC	0.009	0.004	0.006	0.005	0.005
Q23.17	A	0.008	0.005	0.005	0.005	0.006
94UG103	A	0.018	0.015	0.004	0.006	0.006
T257-31	AG	0.072	0.037	0.040	0.041	0.042
211-9	AG	0.038	0.020	0.017	0.017	0.017
620345.c01	AE	>50	>50	>50	>50	>50
BJOX010000.06.2	AE	0.104	0.036	0.029	0.026	0.032
X1632_S2_B10	G	>50	0.008	0.007	0.008	0.009
231965.c01	D	0.633	0.098	0.075	0.090	0.111
89-F1_2_25	CD	>50	>50	>50	>50	>50

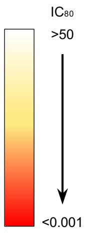

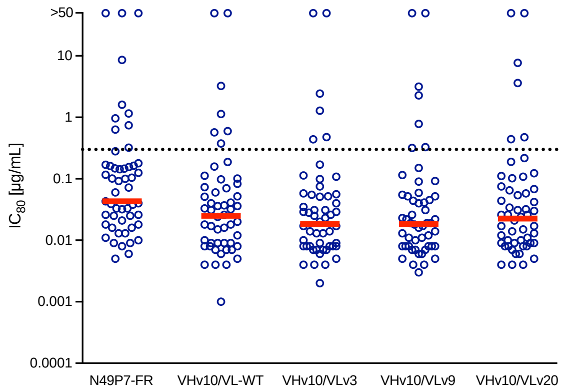

	N49P7-FR	VHv10/VL-WT	VHv10/VLv3	VHv10/VLv9	VHv10/VLv20
Median IC_{80} [µg/mL]	0.043	0.025	0.019	0.019	0.023
GeoMean IC_{80} [µg/mL]	0.067	0.029	0.024	0.025	0.029
Breadth (IC_{80}<0.3) [%]	81	87	88	87	88

Supplementary Fig. 16

HIV pseudovirus	Clade	IC$_{80}$ [µg/mL]				
		N49P7-FR	VH-WT/VLv3	VHv1/VLv3	VHv10/VLv3	VHv13/VLv3
6535.3	B	0.396	0.163	0.010	0.057	0.012
QH0692.42	B	0.223	0.079	0.011	0.049	0.017
SC422661.8	B	0.029	0.011	0.004	0.010	0.005
PVO.4	B	0.116	0.029	0.005	0.032	0.009
AC10.0.29	B	1.938	1.211	0.022	0.174	0.084
RHPA4259.7	B	0.017	0.006	0.005	0.007	0.002
REJO4541.67	B	0.048	0.013	0.005	0.014	0.007
CAAN5342.A2	B	1.407	0.727	0.040	0.473	0.163
WEAU_d15_410_5017	B	0.012	0.005	0.001	0.006	0.004
1006_11_C3_1601	B	0.083	0.020	0.005	0.019	0.007
1054_07_TC4_1499	B	1.174	0.731	0.040	0.383	0.152
1056_10_TA11_1826	B	0.182	0.082	0.007	0.053	0.017
1012_11_TC21_3257	B	0.053	0.015	0.006	0.016	0.006
6240_08_TA5_4622	B	0.538	0.214	0.024	0.222	0.089
6244_13_B5_4576	B	0.128	0.073	0.017	0.047	0.024
62357_14_D3_4589	B	0.223	0.141	0.010	0.089	0.026
SC05_8C11_2344	B	0.109	0.049	0.005	0.022	0.007
Du156.12	C	0.009	0.004	0.004	0.006	0.005
Du172.17	C	0.276	0.134	0.006	0.035	0.014
Du422.1	C	0.500	0.413	0.008	3.573	0.444
ZM197M.PB7	C	0.362	0.201	0.005	0.066	0.022
ZM214M.PL15	C	0.373	0.208	0.024	0.148	0.051
ZM249M.PL1	C	0.013	0.004	0.004	0.008	0.007
ZM53M.PB12	C	2.424	1.552	0.032	0.369	0.164
ZM109F.PB4	C	0.008	0.005	0.005	0.005	0.001
CAP45.2.00.G3	C	1.313	0.356	0.005	0.207	0.140
CAP210.2.00.E8	C	14.190	7.268	0.069	6.276	2.557
HIV-001428-2.42	C	0.005	0.002	0.001	0.004	0.003
HIV-16055-2.3	C	0.057	0.024	0.005	0.015	0.008
Ce0393_C3	C	0.105	0.052	0.007	0.031	0.008
Ce1176_A3	C	0.297	0.256	0.009	0.077	0.019
Ce2010_F5	C	0.084	0.027	0.003	0.018	0.011
Ce703010054_2A2	C	0.449	0.241	0.014	0.054	0.028
246F C1G	C	0.109	0.036	0.004	0.015	0.009
249M B10	C	0.012	0.004	0.003	0.008	0.008
ZM247v1(Rev-)	C	0.041	0.016	0.000	0.010	0.004
7030102001E5(Rev-)	C	0.178	0.145	0.010	0.048	0.021
Ce704809221_1B3	C	0.270	0.119	0.017	0.034	0.022
CNE19	BC	0.063	0.032	0.004	0.013	0.006
CNE21	BC	0.430	0.157	0.007	0.054	0.022
CNE17	BC	0.129	0.069	0.012	0.039	0.017
CNE30	BC	0.391	0.167	0.025	0.087	0.038
CNE52	BC	0.010	0.006	0.005	0.005	0.005
CNE53	BC	0.068	0.034	0.008	0.020	0.010
CNE58	BC	0.023	0.011	0.004	0.009	0.006
BJOX2000	BC	0.179	0.094	0.013	0.050	0.029
CH119	BC	0.217	0.134	0.017	0.064	0.023
Q23.17	A	0.025	0.012	0.005	0.007	0.006
Q461.e2	A	0.139	0.081	0.016	0.027	0.017
0260.v5.c36	A	0.092	0.056	0.013	0.027	0.013
T257-31	AG	0.164	0.092	0.023	0.053	0.029
T250-4	AG	0.274	0.237	0.006	0.011	0.007
T251-18	AG	0.103	0.049	0.012	0.023	0.015
211-9	AG	0.085	0.043	0.029	0.026	0.018
620345.c01	AE	>50	>50	5.655	>50	>50
R2184.c04	AE	0.011	0.006	0.005	0.005	0.005
R3265.c06	AE	0.201	0.113	0.018	0.053	0.035
BJOX009000.02.4	AE	0.094	0.057	0.014	0.028	0.014
BJOX010000.06.2	AE	0.176	0.107	0.020	0.041	0.024
BJOX025000.01.1	AE	0.171	0.064	0.012	0.027	0.017
X1193_C1	G	0.026	0.014	0.006	0.008	0.008
X2088_C9	G	0.051	0.022	0.009	0.019	0.016
X1632_S2_B10	G	>50	2.051	0.011	0.015	0.013
3016.v5.c45	D	0.057	0.030	0.007	0.016	0.009
A07412M1.vrc12	D	0.059	0.065	0.006	0.044	0.021
231965.c01	D	1.121	0.559	0.014	0.117	0.048
3817.v2.c59	CD	1.198	0.771	0.025	0.092	0.040
6811.v7.c18	CD	0.140	0.074	0.015	0.043	0.025
89-F1_2_25	CD	>50	>50	>50	>50	>50
3301.v1.c24	AC	0.042	0.021	0.005	0.012	0.007
6540.v4.c1	AC	0.166	0.255	0.008	0.019	0.005
6545.v4.c1	AC	0.152	0.154	0.008	0.016	0.006
3103.v3.c10	ACD	0.057	0.030	0.013	0.019	0.015

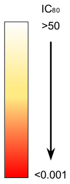

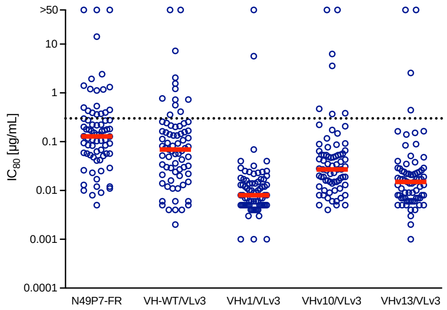

	N49P7-FR	VH-WT/VLv3	VHv1/VLv3	VHv10/VLv3	VHv13/VLv3
Median IC$_{80}$ [µg/mL]	0.129	0.069	0.008	0.027	0.015
GeoMean IC$_{80}$ [µg/mL]	0.126	0.065	0.009	0.033	0.016
Breadth (IC$_{80}$<0.3) [%]	74	84	97	90	95

Supplementary Fig. 17

HIV pseudovirus	Clade	IC$_{80}$ [μg/mL]													
		VRC01	N49P7	N49P7-FR	VHv1/VLv3	VHv1-2/VLv3	VHv1-6/VLv3	VHv1-23/VLv3	VHv1-40/VLv3	VHv1-50/VLv3	VHv1-58/VLv3	VHv1-65/VLv3	VHv1-76/VLv3	VHv1-84/VLv3	VHv1-85/VLv3
6535.3	B	0.696	1.227	0.287	0.050	0.139	0.072	0.107	0.052	0.089	0.128	0.054	0.090	0.086	0.096
PVO.4	B	0.597	0.510	0.092	0.031	0.074	0.063	0.056	0.049	0.078	0.104	0.047	0.064	0.054	0.040
AC10.0.29	B	1.288	>50	1.079	0.114	0.211	0.068	0.094	0.089	0.240	4.832	0.113	0.230	0.235	0.096
RHPA4259.7	B	0.023	0.437	0.019	0.004	0.007	0.009	0.006	0.007	0.009	0.025	0.007	0.008	0.008	0.008
REJO4541.67	B	0.090	0.155	0.024	0.010	0.019	0.016	0.013	0.015	0.015	0.028	0.012	0.019	0.016	0.015
CAAN5342.A2	B	0.686	1.002	2.209	0.164	0.230	0.188	0.282	0.165	0.227	0.416	0.187	0.328	0.302	0.279
WEAU_d15_410_5017	B	0.060	0.093	0.013	0.005	0.013	0.008	0.006	0.007	0.012	0.023	0.007	0.012	0.010	0.009
1006_11_C3_1601	B	0.140	0.188	0.040	0.012	0.021	0.016	0.018	0.015	0.020	0.039	0.014	0.020	0.017	0.018
1054_07_TC4_1499	B	1.011	5.562	1.304	0.149	0.244	0.263	0.281	0.245	0.351	0.726	0.196	0.261	0.218	0.362
1056_10_TA11_1826	B	0.648	0.534	0.109	0.024	0.047	0.032	0.024	0.040	0.026	0.116	0.028	0.039	0.030	0.030
1012_11_TC21_3257	B	0.160	0.162	0.020	0.010	0.033	0.030	0.016	0.024	0.027	0.071	0.020	0.033	0.030	0.018
6240_08_TA5_4622	B	0.955	2.777	0.178	0.060	0.074	0.057	0.054	0.053	0.048	0.117	0.042	0.069	0.049	0.092
6244_13_B5_4576	B	0.183	0.499	0.110	0.045	0.072	0.067	0.080	0.070	0.094	0.133	0.065	0.057	0.062	0.081
62357_14_D3_4589	B	0.796	0.199	0.115	0.047	0.058	0.046	0.061	0.037	0.059	0.078	0.037	0.054	0.043	0.060
SC05_8C11_2344	B	0.313	0.306	0.089	0.020	0.048	0.039	0.032	0.041	0.036	0.067	0.039	0.038	0.038	0.030
Du422.1	C	>50	>50	0.343	0.033	0.076	0.029	0.030	0.029	0.048	0.095	0.028	0.060	0.057	0.033
ZM197M.PB7	C	0.346	0.613	0.306	0.014	0.036	0.027	0.021	0.031	0.031	0.069	0.018	0.033	0.025	0.021
ZM53M.PB12	C	1.146	1.244	1.608	0.085	0.114	0.058	0.095	0.082	0.076	0.123	0.061	0.095	0.070	0.102
ZM109F.PB4	C	0.094	0.233	0.012	0.003	0.020	0.022	0.006	0.014	0.011	0.037	0.009	0.013	0.011	0.004
CAP45.2.00.G3	C	3.388	0.554	1.113	0.028	7.593	0.023	0.019	0.034	12.792	16.337	0.019	3.358	4.020	0.014
CAP210.2.00.E8	C	>50	3.546	8.313	0.172	1.989	0.320	0.177	0.242	3.828	1.080	0.226	2.393	1.859	0.204
HIV-16055-2.3	C	0.092	0.372	0.034	0.008	0.013	0.011	0.006	0.009	0.014	0.028	0.008	0.013	0.011	0.011
Ce0393_C3	C	0.316	0.259	0.113	0.016	0.032	0.019	0.014	0.014	0.029	0.064	0.013	0.034	0.023	0.023
Ce1176_A3	C	1.844	0.897	0.359	0.058	0.063	0.056	0.055	0.052	0.127	0.184	0.060	0.062	0.049	0.060
Ce2010_F5	C	0.229	0.542	0.078	0.009	0.009	0.010	0.006	0.009	0.012	0.028	0.008	0.009	0.010	0.011
Ce703010054_2A2	C	0.669	0.406	0.354	0.037	0.103	0.114	0.082	0.132	0.066	0.440	0.071	0.070	0.057	0.078
246F C1G	C	1.907	0.165	0.038	0.007	0.033	0.490	0.040	0.183	0.045	>50	0.157	0.031	0.026	0.019
249M B10	C	0.064	0.343	0.013	0.006	0.014	0.012	0.010	0.011	0.013	0.072	0.010	0.012	0.010	0.009
ZM247v1(Rev-)	C	0.318	0.272	0.045	0.003	>50	0.043	0.022	0.035	>50	>50	0.022	>50	>50	0.015
7030102001E5(Rev-)	C	0.501	0.399	0.165	0.016	0.035	0.029	0.024	0.018	0.026	0.070	0.024	0.027	0.023	0.026
Ce704809221_1B3	C	0.414	2.228	0.233	0.020	0.059	0.044	0.041	0.046	0.041	0.192	0.023	0.052	0.036	0.045
CNE21	BC	0.189	37.177	0.424	0.016	0.044	0.016	0.017	0.013	0.032	0.064	0.020	0.029	0.026	0.012
CNE17	BC	0.570	0.459	0.097	0.017	0.032	0.028	0.019	0.028	0.033	0.059	0.019	0.032	0.027	0.031
CNE53	BC	0.090	0.131	0.030	0.006	0.011	0.009	0.005	0.009	0.010	0.026	0.008	0.009	0.010	0.011
CNE58	BC	0.143	0.088	0.010	0.004	0.005	0.005	0.002	0.005	0.005	0.008	0.004	0.005	0.005	0.005
BJOX2000	BC	>50	3.839	0.121	0.017	0.125	0.018	0.015	0.019	0.073	0.174	0.014	0.179	0.125	0.018
Q23.17	A	0.047	0.203	0.010	0.004	0.008	0.007	0.004	0.006	0.007	0.021	0.005	0.008	0.007	0.006
Q461.e2	A	0.363	0.922	0.055	0.016	0.051	0.046	0.031	0.039	0.050	0.205	0.033	0.058	0.045	0.036
0260.v5.c36	A	0.304	0.854	0.121	0.024	0.070	0.062	0.046	0.057	0.078	0.212	0.047	0.085	0.059	0.049
T250-4	AG	>50	7.517	2.190	0.006	0.012	0.136	0.029	0.419	0.015	0.033	0.014	0.012	0.013	0.092
T251-18	AG	1.937	0.407	0.069	0.013	0.050	0.038	0.018	0.039	0.044	0.119	0.027	0.053	0.033	0.022
211-9	AG	8.585	0.419	0.052	0.028	0.066	0.074	0.048	0.073	0.096	0.279	0.052	0.088	0.059	0.042
620345.c01	AE	>50	>50	>50	43.540	>50	>50	>50	>50	>50	>50	>50	>50	>50	>50
R2184.c04	AE	0.089	0.039	0.007	0.005	0.011	0.010	0.006	0.008	0.011	0.026	0.007	0.011	0.010	0.006
R3265.c06	AE	0.346	0.992	0.109	0.020	0.042	0.028	0.016	0.026	0.040	0.097	0.022	0.042	0.030	0.036
BJOX009000.02.4	AE	1.036	1.142	0.095	0.025	0.170	0.143	0.075	0.117	0.136	0.273	0.082	0.150	0.102	0.076
X1193_C1	G	0.081	0.126	0.013	0.006	0.014	0.012	0.006	0.010	0.013	0.034	0.008	0.013	0.015	0.009
X2088_C9	G	>50	0.307	0.019	0.010	0.019	0.019	0.011	0.014	0.024	0.052	0.014	0.023	0.020	0.017
A07412M1.vrc12	D	0.054	0.831	0.258	0.005	0.025	0.029	0.017	0.023	0.033	0.056	0.017	0.030	0.019	0.033
3817.v2.c59	CD	>50	35.523	2.290	0.057	0.297	1.148	0.224	0.344	0.349	1.197	0.441	0.334	0.411	0.198
6811.v7.c18	CD	0.110	1.201	0.116	0.017	0.098	0.048	0.027	0.039	0.103	0.229	0.024	0.080	0.068	0.034
89-F1_2_25	CD	>50	>50	>50	>50	>50	3.206	2.763	0.625	>50	>50	3.555	>50	>50	4.931
3301.v1.c24	AC	0.154	0.062	0.017	0.005	0.010	0.009	0.006	0.009	0.010	0.014	0.007	0.010	0.009	0.008
6540.v4.c1	AC	>50	>50	0.289	0.006	>50	0.011	0.003	0.012	>50	>50	0.002	>50	>50	0.003
6545.v4.c1	AC	>50	>50	0.176	0.020	>50	0.024	0.005	0.010	>50	>50	0.002	>50	>50	0.003
3103.v3.c10	ACD	0.799	0.204	0.046	0.022	1.113	5.712	0.767	3.903	1.263	>50	2.699	0.994	0.560	0.189

IC$_{80}$
>50
<0.001

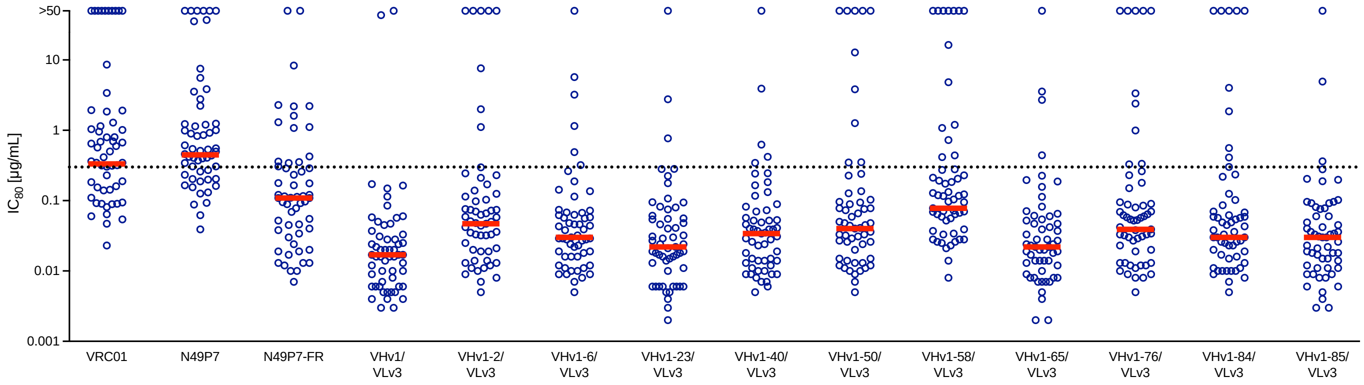

	VRC01	N49P7	N49P7-FR	VHv1/VLv3	VHv1-2/VLv3	VHv1-6/VLv3	VHv1-23/VLv3	VHv1-40/VLv3	VHv1-50/VLv3	VHv1-58/VLv3	VHv1-65/VLv3	VHv1-76/VLv3	VHv1-84/VLv3	VHv1-85/VLv3
Median IC$_{80}$ [μg/mL]	0.332	0.448	0.109	0.017	0.047	0.030	0.022	0.034	0.040	0.078	0.022	0.039	0.030	0.030
GeoMean IC$_{80}$ [μg/mL]	0.328	0.563	0.110	0.019	0.050	0.042	0.026	0.036	0.048	0.104	0.028	0.047	0.040	0.030
Breadth (IC$_{80}$<0.3) [%]	34	29	73	96	86	89	95	91	82	75	93	82	82	95

Supplementary Fig. 18

HIV pseudovirus	Clade	IC$_{80}$ [µg/mL]						
		VRC01	N49P7	N49P7-FR	VHv1/VLv3	VHv1-23/VLv3	VHv1-65/VLv3	VHv1-85/VLv3
H704_0726_080sN	B	6.826	1.112	0.196	0.042	0.055	0.059	0.079
H704_1535_030sN	B	0.060	0.162	0.023	0.004	0.005	0.005	0.005
H704_0911_150sN	B	1.118	0.564	3.177	0.167	0.321	0.282	0.423
H704_0907_130sN	B	0.240	0.218	0.026	0.006	0.012	0.019	0.012
H704_1429_090_eN1A	B	0.131	3.646	0.217	0.005	0.009	0.012	0.010
H704_1429_090_eN2G	B	0.148	>50	0.329	0.006	0.012	0.012	0.013
H704_0496_040sN	B	2.974	0.119	0.099	0.006	0.018	0.025	0.013
H704_2788_060eN_12	B	1.298	0.129	0.089	0.010	0.016	0.016	0.019
H704_2767_070sN	B	3.217	>50	2.711	0.008	>50	>50	>50
H704_2788_060eN_04	B	1.719	0.170	0.121	0.012	0.019	0.019	0.025
H704_0644_060sN_prelimSeq	B	0.471	0.326	0.148	0.021	0.032	0.026	0.056
H704_0847_030_EsN_02C	B	3.828	0.227	0.151	0.019	0.076	0.110	0.072
H704_1775_030cN_SynGtoA_567	B	0.095	0.374	0.086	0.008	0.022	0.022	0.023
H704_0847_030_EsN_01T	B	0.958	0.516	0.072	0.015	0.048	0.069	0.119
H704_0855_080_EsN	B	0.011	0.107	0.004	0.003	0.002	0.001	0.002
H704_0575_060_EsN1A	B	2.015	1.308	0.740	0.046	0.091	0.110	0.108
H704_0575_060_EsN2T	B	1.731	1.341	0.689	0.052	0.096	0.094	0.158
H704_3008_040EsN	B	3.190	0.980	0.134	0.005	0.042	0.669	0.056
H704_0513_150_eN01T	B	1.987	0.847	0.487	0.062	0.074	0.070	0.176
H704_1183_220EsN	B	0.265	0.111	0.010	0.006	0.009	0.011	0.009
H704_1180_070EsN	B	0.069	0.096	0.006	0.009	0.007	0.008	0.011
H704_2541_080EsN	B	0.085	0.116	0.016	0.012	0.009	0.007	0.011
H704_2448_240_RE_cs	B	0.057	0.670	0.029	0.011	0.016	0.016	0.024
H704_1783_150_RE_cs	B	0.085	0.097	0.016	0.006	0.007	0.008	0.007
H704_2095_130_RE_cs	B	0.229	0.208	0.028	0.008	0.015	0.014	0.018
H704_0944_180_RE_cs	B	0.074	0.068	0.007	0.001	0.005	0.006	0.005
H704_2839_140_RE_cs	B	1.636	0.142	0.173	0.011	0.016	0.011	0.012
H704_1109_140_RE_cs	B	3.559	5.596	8.196	0.077	0.622	0.635	0.297
H704_0575_060_RE_p002s	B	1.979	0.931	0.770	0.041	0.050	0.086	0.076
H704_0746_760_RE_p001s	B	1.308	0.728	0.268	0.029	0.058	0.074	0.087
H704_0746_760_RE_p002s	B	0.993	0.719	0.233	0.030	0.054	0.074	0.088
H704_0746_760_RE_p003s	B	0.921	0.692	0.270	0.028	0.066	0.074	0.093
H704_1991_230_RE_p001s_1194T	B	1.376	0.575	0.245	0.020	0.052	0.048	0.055
H704_1991_230_RE_p002s_1194T	B	1.669	0.557	0.203	0.021	0.039	0.040	0.047
H704_2065_060_RE_p001s	B	0.206	0.705	0.067	0.042	0.047	0.039	0.043
H704_2065_060_RE_p002s	B	0.136	0.565	0.171	0.025	0.037	0.042	0.050
H704_2541_080_RE_p001s	B	0.138	0.130	0.019	0.009	0.010	0.010	0.012
H704_2684_181_RE_p001s	B	0.112	0.135	0.025	0.010	0.010	0.011	0.011
H704_2981_150_RE_p001s_2559A	B	3.510	8.678	3.304	0.332	0.590	0.651	0.673
H704_2981_150_RE_p002s_2559A	B	1.167	3.096	1.180	0.122	0.175	0.228	0.218
H704_1930_170_RE_c01s_1523A	B	0.100	0.108	0.042	0.012	0.010	0.010	0.015
H704_1930_170_RE_c02s_1523G	B	0.207	0.123	0.072	0.021	0.015	0.017	0.023
H704_0445_180_RE_con_s	B	>50	>50	>50	0.041	0.098	4.390	0.138
H704_2555_240_RE_con_s_vpuG	B	0.412	0.108	0.073	0.021	0.022	0.022	0.032
H704_2834_210_RE_pb001_s	B	1.957	0.517	0.215	0.033	0.047	0.077	0.058
H704_2834_210_RE_pb002_s	B	2.593	0.484	0.293	0.049	0.065	0.111	0.075
H704_0128_220_RE_pb001_s	B	1.552	0.448	0.405	0.010	0.015	0.017	0.022
H704_0856_240_RE_pb001_s	B	0.649	0.649	0.168	0.053	0.056	0.053	0.107
H704_0886_250_RE_p001s	B	1.540	0.659	0.433	0.042	0.076	0.062	0.087
H704_2541_080_RE_p003s	B	0.155	0.105	0.020	0.018	0.010	0.011	0.015
H704_0026_231_RE_pbsga001_s	B	0.407	0.477	0.454	0.015	0.024	0.022	0.031
H704_0513_150_RE_pblib003_s	B	1.234	0.484	0.271	0.061	0.064	0.074	0.096
H704_1180_070_RE_pblib027_s	B	0.111	0.073	0.009	0.020	0.008	0.010	0.015
H704_1528_240_RE_pblib_001_s	B	2.006	0.132	0.046	0.014	0.016	0.032	0.019
H704_2684_181_RE_pblib006_s	B	0.091	0.096	0.018	0.007	0.007	0.010	0.009
H704_2981_150_RE_pblib003_s	B	1.342	1.351	0.661	0.061	0.068	0.186	0.143
H704_2981_150_RE_pblib005_s	B	1.461	0.994	0.202	0.029	0.061	0.121	0.094
H704_3000_240_RE_pbsga002_s	B	0.639	0.195	0.940	0.017	0.022	0.020	0.026
V704_0372_250_RE_pblib001_s	B	0.357	0.161	0.051	0.007	0.010	0.018	0.020
H704_3000_240_RE_pbsga001_s	B	0.914	0.161	0.482	0.015	0.021	0.021	0.024
V704_0372_250_RE_pblib002_s	B	0.254	0.098	0.031	0.006	0.007	0.013	0.012

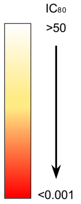

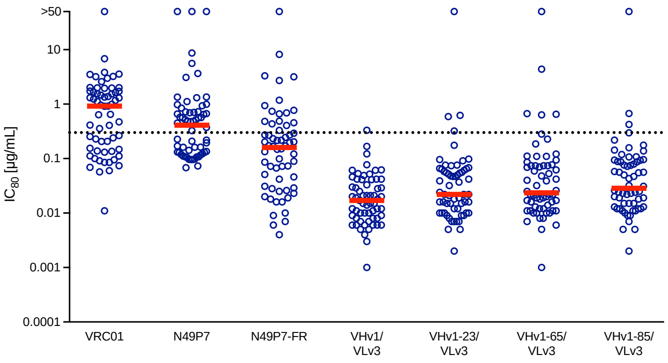

	VRC01	N49P7	N49P7-FR	VHv1/VLv3	VHv1-23/VLv3	VHv1-65/VLv3	VHv1-85/VLv3
Median IC$_{80}$ [µg/mL]	0.918	0.411	0.160	0.017	0.022	0.024	0.029
GeoMean IC$_{80}$ [µg/mL]	0.544	0.355	0.127	0.018	0.027	0.035	0.035
Breadth (IC$_{80}$<0.3) [%]	38	44	72	98	93	92	95

Supplementary Fig. 19

HIV pseudovirus	Clade	IC$_{80}$ [µg/mL]						
		VRC01	N49P7	N49P7-FR	VHv1/VLv3	VHv1-23/VLv3	VHv1-65/VLv3	VHv1-85/VLv3
H703_0109_210s	C	1.091	1.403	0.193	0.022	0.087	0.134	0.062
H703_0309_100s	C	0.314	0.484	0.507	0.010	0.025	0.019	0.025
V703_0309_100_RE_pblib002_s	C	0.203	0.276	0.328	0.008	0.017	0.015	0.019
H703_0322_130s_M1I	C	0.854	0.311	0.128	0.012	0.043	0.049	0.063
V703_0597_190_RE_pblib002_s	C	0.967	0.833	0.050	0.010	0.060	0.176	0.040
V703_0597_190_RE_pblib003_s	C	0.462	0.728	0.066	0.014	0.066	0.154	0.043
H703_0597_190_RE_e5A1s	C	0.478	0.657	0.041	0.010	0.049	0.148	0.037
H703_0636_200Es	C	0.272	0.123	0.016	0.006	0.014	0.010	0.014
V703_0712_250_RE_pblib001_s	C	1.526	0.213	0.168	0.009	0.020	0.026	0.018
V703_0712_250_RE_pblib002_s	C	0.139	0.093	0.057	0.004	0.007	0.008	0.007
H703_0860_150Es	C	3.4070	0.115	0.252	0.005	0.008	0.013	0.010
H703_0967_040s	C	1.2860	0.027	0.049	0.003	0.006	0.005	0.005
H703_1026_120Es_A5	C	10.3600	0.715	0.182	0.015	0.047	0.076	0.052
H703_1687_100Es	C	3.8020	0.397	0.189	0.013	0.084	0.720	0.066
H703_1828_220Es	C	1.4640	0.123	0.033	0.004	0.009	0.011	0.009
V703_2141_160_RE_sga3D6_s	C	8.9160	15.440	0.206	0.009	0.130	0.657	0.043
V703_3000_090_RE_pblib002_s	C	1.6330	0.261	0.029	0.003	0.008	0.014	0.011
H703_0795_040s	C	4.9490	0.682	0.058	0.015	0.054	0.083	0.059
V703_1383_240_RE_pblib002_s	C	0.5237	0.066	0.012	0.002	0.007	0.008	0.006
H703_1383_240_RE_e5D3s	C	0.9610	0.086	0.015	0.004	0.007	0.008	0.007
V703_1383_240_RE_pblib003_s	C	0.4725	0.047	0.009	0.004	0.005	0.006	0.005
H703_1407_090s_4G4	C	5.2500	0.229	0.075	0.036	0.029	0.026	0.038
V703_1407_090_RE_pblib003_s	C	2.0780	0.248	0.060	0.013	0.019	0.019	0.023
H703_1453_240_RE_e9B7s	C	8.0940	0.168	0.153	0.013	0.031	0.036	0.033
H703_1714_080c	C	0.6877	12.840	0.305	0.003	0.006	0.005	0.005
H703_1758_260_RE_cs	C	0.8127	0.323	0.067	0.012	0.025	0.023	0.029
H703_1783_170Es	C	3.3070	0.304	0.074	0.006	0.012	0.009	0.014
V703_1855_162_RE_pblib003_s	C	2.4070	0.237	0.342	0.008	0.020	0.008	0.008
H703_1945_090s_2A3	C	1.9830	0.139	0.035	0.002	0.007	0.007	0.006
H703_1945_090s_2F1	C	0.7662	0.160	0.035	0.015	0.025	0.025	0.021
H703_1945_090s_3D5	C	1.950	0.075	0.026	0.020	0.026	0.041	0.028
V703_2372_170_RE_con_s	C	3.8890	0.183	0.074	0.009	0.012	0.020	0.013

IC$_{80}$
>50
<0.001

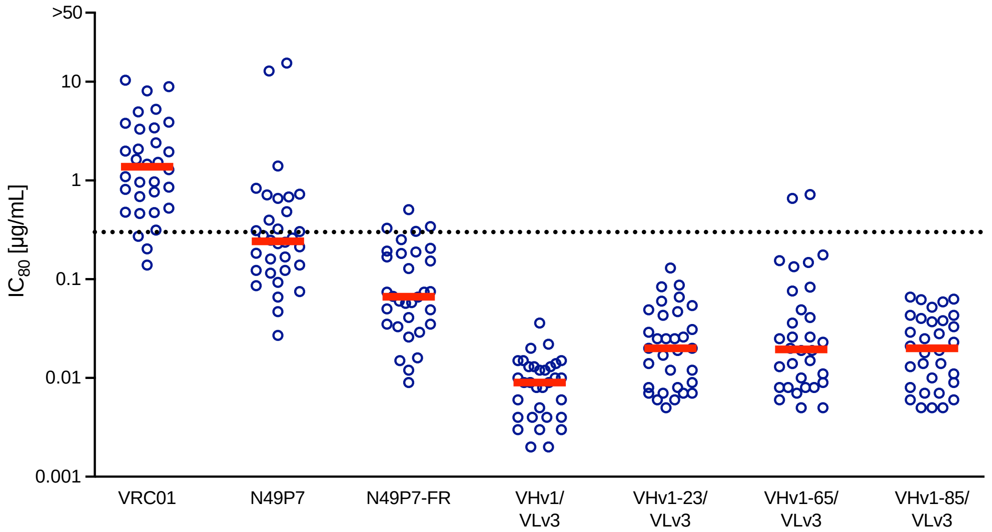

	VRC01	N49P7	N49P7-FR	VHv1/VLv3	VHv1-23/VLv3	VHv1-65/VLv3	VHv1-85/VLv3
Median IC$_{80}$ [µg/mL]	1.375	0.243	0.067	0.009	0.020	0.020	0.020
GeoMean IC$_{80}$ [µg/mL]	1.349	0.286	0.074	0.008	0.020	0.027	0.019
Breadth (IC$_{80}$<0.3) [%]	9	59	88	100	100	94	100

Supplementary Fig. 20

HIV pseudovirus	Clade	IC80 [μg/mL]			
		VRC01	N49P7	N49P7-FR	VHv1-23/VLv3
6535.3	B	5.672	1.227	1.662	0.312
QH0692.42	B	4.207	4.017	0.342	0.287
SC422661.8	B	0.367	0.526	0.074	0.048
PVO.4	B	1.336	0.510	0.130	0.096
TRO.11	B	1.251	0.302	0.080	0.046
AC10.0.29	B	4.317	>50	16.531	0.761
RHPA4259.7	B	0.157	0.437	0.092	0.036
THRO4156.18	B	17.228	26.972	38.453	2.846
REJO4541.67	B	0.245	0.155	0.079	0.044
TRJO4551.58	B	0.268	0.689	0.200	0.044
WITO4160.33	B	0.381	0.448	0.078	0.028
CAAN5342.A2	B	3.256	3.380	4.198	0.719
WEAU_d15_410_787	B	0.277	0.093	0.066	0.026
1006_11_C3_1601	B	0.530	0.188	0.137	0.053
1054_07_TC4_1499	B	3.741	5.562	4.223	0.927
1056_10_TA11_1826	B	2.956	0.534	0.371	0.100
1012_11_TC21_3257	B	0.387	0.162	0.076	0.064
6240_08_TA5_4622	B	2.281	2.777	0.903	0.187
6244_13_B5_4576	B	0.535	0.499	0.310	0.182
62357_14_D3_4589	B	5.208	0.344	0.248	0.064
SC05_8C11_2344	B	1.829	0.759	0.321	0.127
Du156.12	C	0.249	0.159	0.050	0.026
Du172.17	C	>50	1.118	0.657	0.049
Du422.1	C	>50	>50	0.648	0.058
ZM197M.PB7	C	1.643	0.613	1.069	0.071
ZM214M.PL15	C	3.422	1.254	0.283	0.182
ZM233M.PB6	C	10.873	0.859	0.816	>50
ZM249M.PL1	C	0.273	0.150	0.052	0.050
ZM53M.PB12	C	3.311	1.244	2.334	0.273
ZM109F.PB4	C	0.476	0.233	0.036	0.036
ZM135M.PL10a	C	4.658	2.552	6.379	0.157
CAP45.2.00.G3	C	24.728	0.554	6.962	0.057
CAP210.2.00.E8	C	>50	7.503	34.836	0.913
HIV-001428-2.42	C	0.052	0.024	0.012	0.006
HIV-0013095-2.11	C	0.294	0.382	0.189	0.054
HIV-16055-2.3	C	0.288	0.372	0.082	0.046
HIV-16845-2.22	C	13.069	6.738	2.013	0.549
Ce1086_B2	C	1.772	0.463	0.526	0.123
Ce0393_C3	C	2.512	0.316	0.084	0.174
Ce1176_A3	C	6.696	0.897	0.980	0.140
Ce2010_F5	C	1.323	0.542	0.278	0.057
Ce0682_E4	C	0.723	0.275	0.079	0.019
Ce1172_H1	C	>50	>50	>50	0.127
Ce2060_G9	C	1.171	2.056	0.792	0.309
Ce703010054_2A2	C	1.519	0.406	0.437	0.203
BF1266.431a	C	0.207	0.077	0.060	0.026
246F C1G	C	0.743	0.165	0.234	0.313
249M B10	C	0.275	0.343	0.074	0.097
ZM247v1(Rev-)	C	1.122	0.272	0.115	0.066
7030102001E5(Rev-)	C	2.463	0.865	0.438	0.005
1394C9G1(Rev-)	C	1.707	>50	>50	0.359
Ce704809221_1B3	C	2.120	2.228	0.852	0.179
CNE19	BC	0.750	0.072	0.108	0.010
CNE20	BC	28.358	0.359	0.182	0.039
CNE21	BC	1.265	37.177	3.270	0.043
CNE17	BC	1.909	0.704	0.410	0.129
CNE30	BC	2.569	0.542	0.625	0.132
CNE52	BC	0.580	0.046	0.012	0.018
CNE53	BC	0.307	0.131	0.160	0.057
CNE58	BC	0.490	0.088	0.047	0.030
MS208.A1	A	0.690	0.353	0.094	0.055
Q23.17	A	0.235	0.203	0.046	0.029
Q461.e2	A	1.530	0.922	0.117	0.128
Q769.d22	A	0.106	0.170	0.048	0.038
Q259.d2.17	A	0.271	33.009	0.050	0.045
Q842.d12	A	0.065	0.075	0.010	0.017
0260.v5.c36	A	1.488	0.854	0.195	0.175
3415.v1.c1	A	0.229	0.508	0.036	0.082
3365.v2.c2	A	0.165	0.137	0.043	0.026
191955_A11	A	2.877	>50	9.224	0.024
191084 B7-19	A	0.392	0.179	0.057	0.052
9004SS_A3_4	A	1.750	0.540	0.111	0.078
T257-31	AG	6.664	0.892	0.492	0.243
928-28	AG	1.313	0.676	0.141	0.173
263-8	AG	0.592	0.301	0.047	0.038
T250-4	AG	>50	>50	7.483	0.097
T251-18	AG	10.515	0.475	0.206	0.068
T278-50	AG	>50	>50	>50	>50
T255-34	AG	1.630	0.272	0.104	0.037
211-9	AG	>50	1.112	0.108	0.287
235-47	AG	0.167	0.191	0.027	0.019
620345.c01	AE	>50	>50	>50	>50
CNE8	AE	1.337	0.334	0.087	0.030
C1080.c03	AE	9.360	1.899	0.452	0.197
R2184.c04	AE	0.330	0.057	0.015	0.028
R1166.c01	AE	4.695	0.505	0.074	0.062
R3265.c06	AE	1.949	0.597	0.219	0.147
C2101.c01	AE	0.713	0.620	0.198	0.064
C3347.c11	AE	0.325	0.093	0.053	0.057
C4118.c09	AE	0.576	0.247	0.202	0.051
CNE5	AE	0.932	0.517	0.962	0.190
BJOX009000.02.4	AE	5.184	1.142	0.364	0.573
BJOX015000.11.5	AE	0.242	0.565	0.163	0.063
BJOX010000.06.2	AE	17.641	0.756	0.323	0.532
BJOX025000.01.1	AE	33.854	1.100	0.138	0.141
BJOX028000.10.3	AE	0.958	0.552	0.065	0.031
X1193_c1	G	0.407	0.126	0.056	0.049
P0402_c2_11	G	0.455	0.210	0.106	0.086
X1254_c3	G	0.157	>50	0.616	0.273
X2088_c9	G	>50	1.007	0.068	0.148
X2131_C1_B5	G	1.335	0.485	0.222	0.242
P1981_C5_3	G	0.916	0.419	0.360	0.376
X1632_S2_B10	G	0.679	>50	>50	38.264
3016.v5.c45	D	0.356	7.934	0.122	0.077
A07412M1.vrc12	D	0.499	0.831	0.728	0.142
231965.c01	D	1.217	0.413	2.187	0.182
231966.c02	D	0.523	0.892	0.145	0.058
6405.v4.c34	D	6.039	0.543	1.344	0.202
3817.v2.c59	CD	>50	35.523	12.494	3.744
6480.v4.c25	CD	0.076	0.166	>50	0.041
6952.v1.c20	CD	0.137	>50	0.204	0.111
6811.v7.c18	CD	0.513	1.201	>50	0.247
89-F1_2_25	CD	>50	>50	>50	>50
3301.v1.c24	AC	0.310	0.060	0.017	0.028
6041.v3.c23	AC	0.085	3.200	>50	0.019
6540.v4.c1	AC	>50	>50	4.031	4.480
6545.v4.c1	AC	>50	>50	0.015	0.032
0815.v3.c3	ACD	0.102	0.051	0.078	0.021
3103.v3.c10	ACD	3.634	0.425	0.046	4.713

IC80 >50 → <0.001

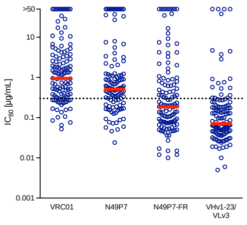

	VRC01	N49P7	N49P7-FR	VHv1-23/VLv3
Median IC80 [μg/mL]	0.945	0.508	0.186	0.071
GeoMean IC80 [μg/mL]	1.008	0.540	0.240	0.098
Breadth (IC80<0.3) [%]	20	26	57	82

Supplementary Fig. 21

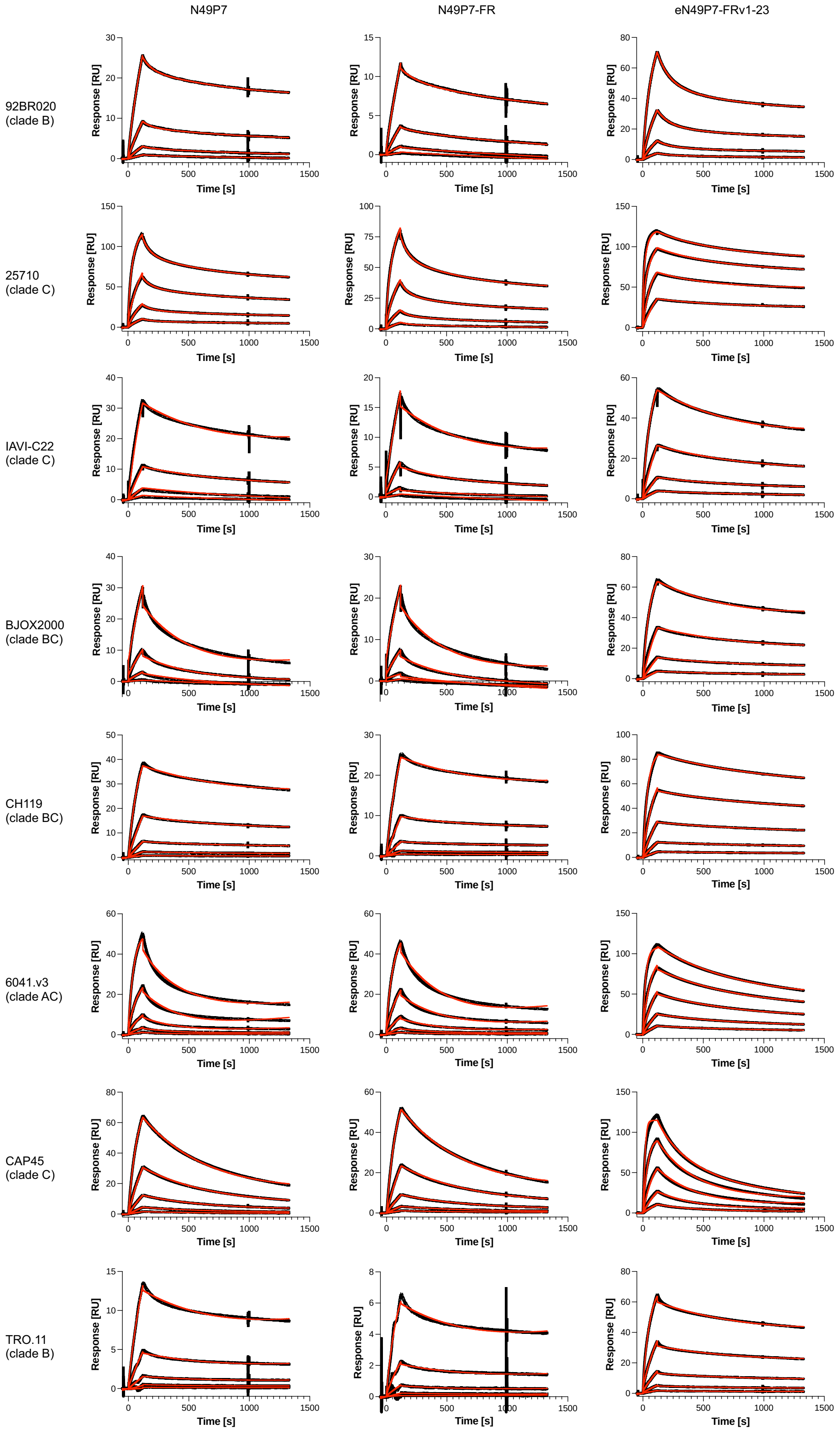

Supplementary Fig. 22

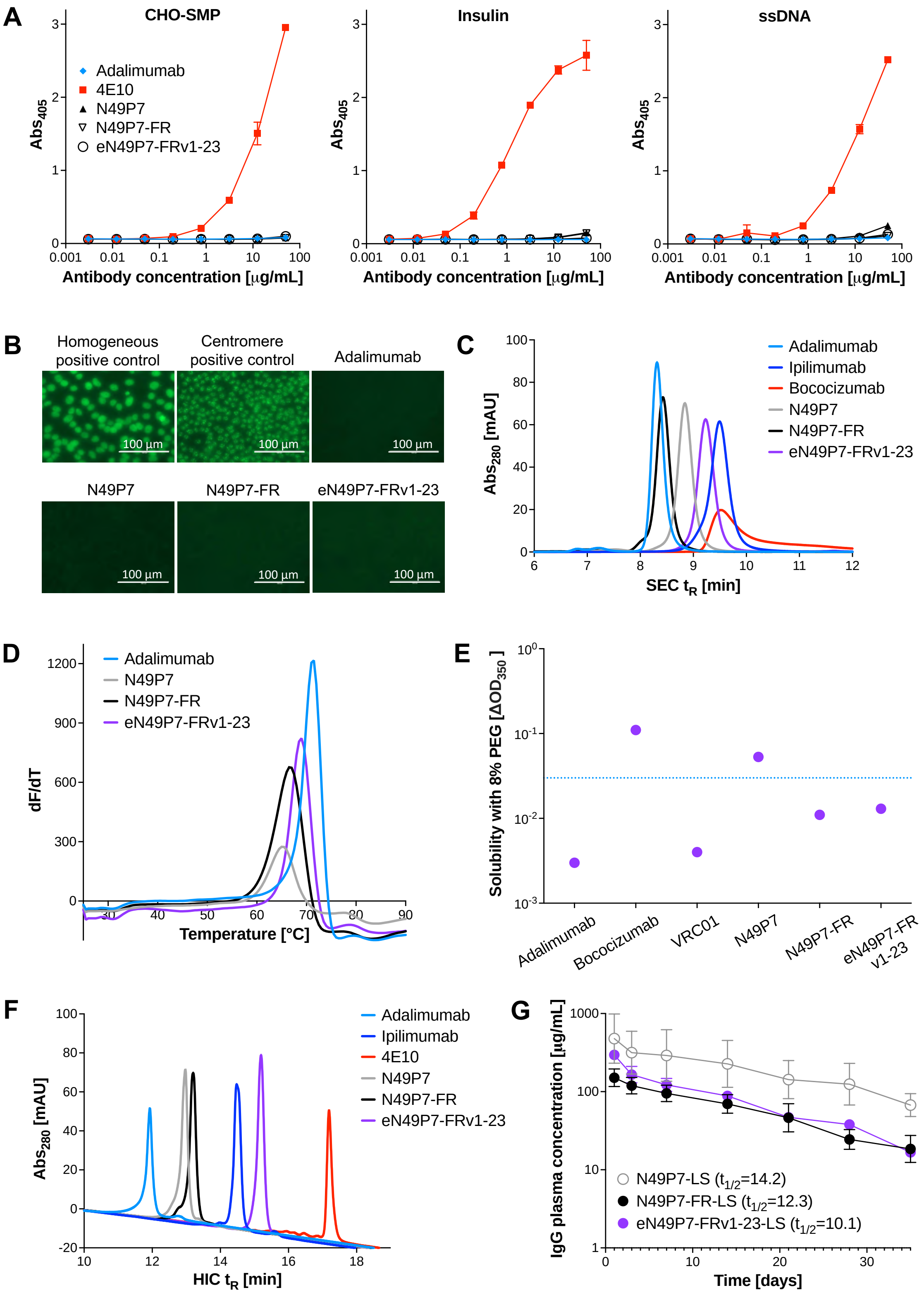

Supplementary Fig. 23

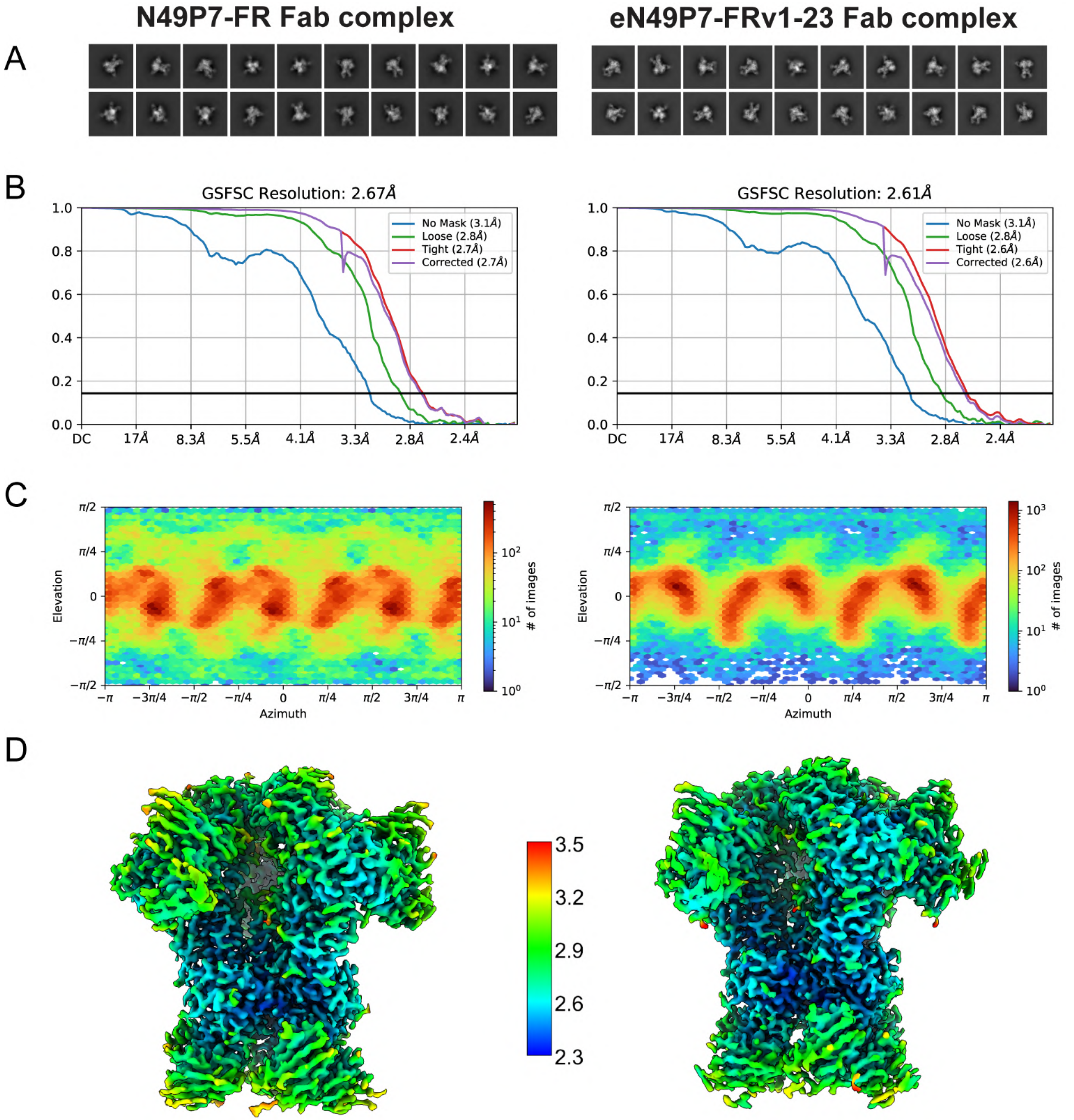

Supplementary Fig. 24

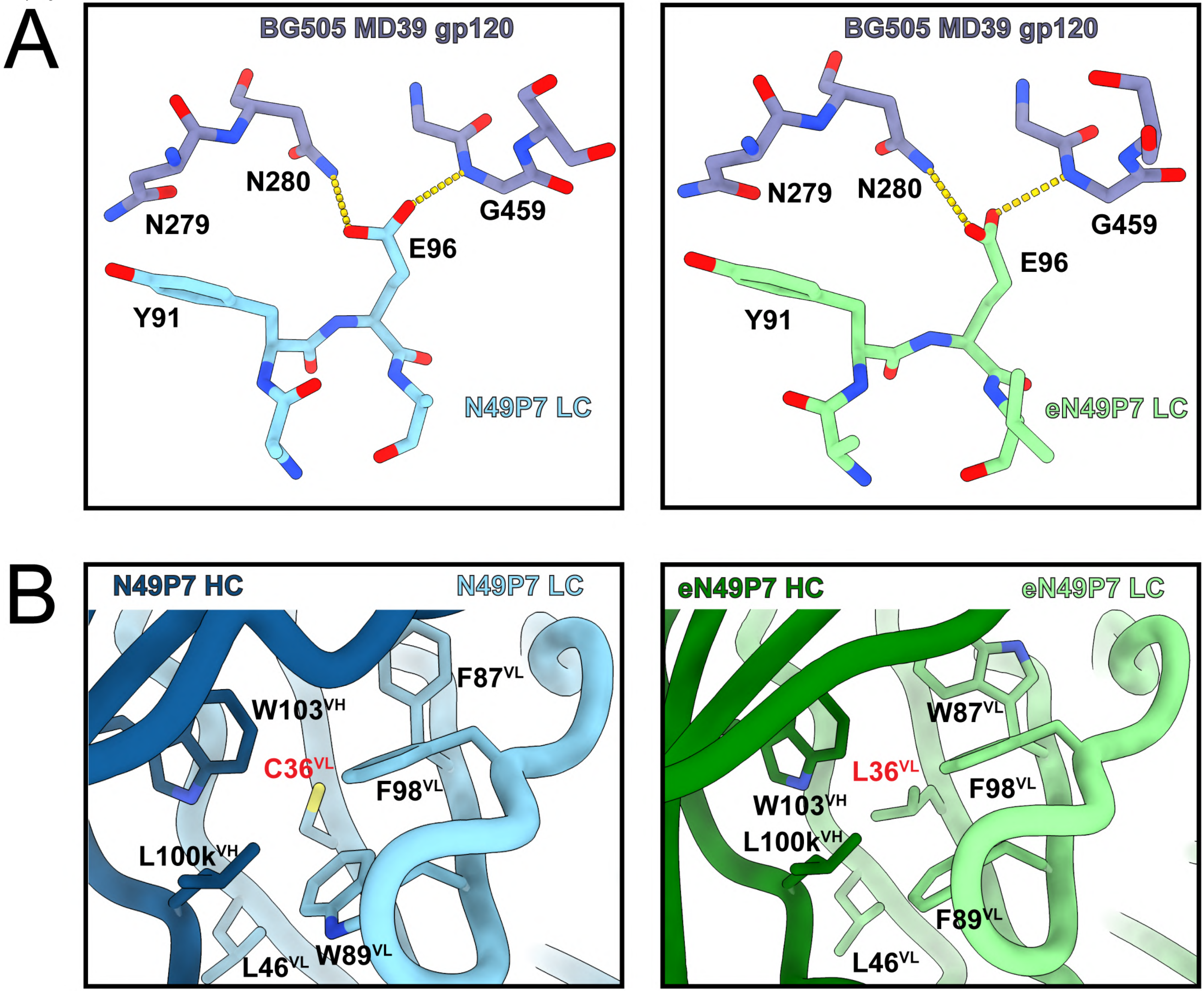

Supplementary Fig. 25

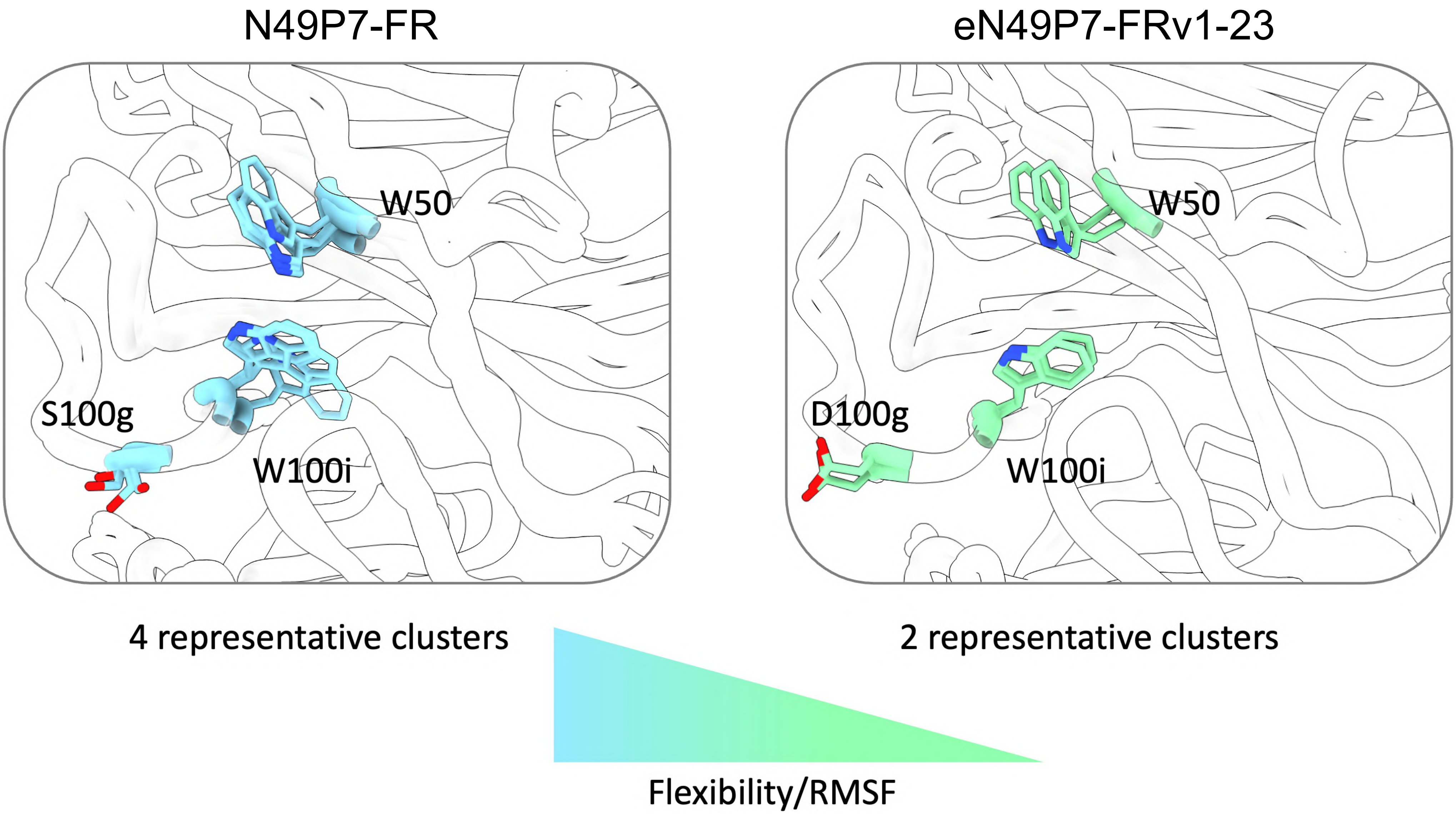

bioRxiv preprint doi: https://doi.org/10.1101/2025.10.22.684031; this version posted October 23, 2025. The copyright holder for this preprint (which was not certified by peer review) is the author/funder, who has granted bioRxiv a license to display the preprint in perpetuity. It is made available under aCC-BY 4.0 International license.

Supplementary Table 1. Most enriched combinatorial variants of N49P7-FR VH reformatted as IgG1 and evaluated for functionality and developability. For each variant, only the sampled amino acids are shown, ordered by position and numbered according to the Kabat scheme.

N49P7-FR variant	Amino acid position															
	**6**	**30**	**33**	**53**	**54**	**56**	**57**	**73**	**74**	**75**	**76**	**76a**	**76b**	**76d**	**100**	**100c**
**VH WT**	Q	P	I	M	G	Q	V	L	S	Q	D	P	D	P	S	R
**VHv1**	Q	T	F	M	Y	Q	M	R	S	W	D	P	Y	P	Y	H
**VHv2**	S	R	F	R	W	Q	V	L	W	W	D	P	D	P	S	R
**VHv3**	Q	R	F	R	W	Q	V	L	W	W	D	P	D	P	S	R
**VHv4**	S	T	I	R	F	Q	V	L	S	Q	D	R	D	R	Y	T
**VHv5**	S	T	F	M	Y	Q	M	R	S	W	D	P	Y	P	Y	H
**VHv6**	S	R	F	R	W	Q	V	L	W	W	D	P	D	P	Y	H
**VHv7**	Q	R	F	M	Y	Q	M	R	S	Q	W	P	Y	R	S	L
**VHv8**	S	P	I	R	H	Q	V	R	S	Q	D	W	D	P	Y	H
**VHv9**	S	R	F	R	W	Q	V	L	W	W	D	P	D	P	S	L
**VHv10**	Q	T	F	M	Y	Q	M	L	W	W	D	P	D	P	S	R
**VHv11**	Q	R	F	M	Y	Q	M	R	S	Q	W	P	Y	R	S	H
**VHv12**	S	T	I	R	H	Q	V	R	S	Q	D	W	D	P	Y	H
**VHv13**	Q	P	F	R	W	Q	V	L	W	W	D	P	D	P	S	R
**VHv14**	S	R	F	R	W	Q	V	L	W	W	D	P	D	P	S	T
**VHv15**	S	R	F	M	Y	Q	M	R	S	Q	W	P	Y	R	S	L
**VHv16**	Q	R	F	R	W	Q	V	L	W	W	D	P	D	P	S	L
**VHv17**	S	R	F	R	W	Q	V	L	W	W	D	P	D	P	Y	T
**VHv18**	S	T	I	R	W	Q	V	L	W	W	D	P	D	P	S	R
**VHv19**	Q	T	F	R	W	Q	V	L	W	W	D	P	D	P	S	R
**VHv20**	S	T	F	R	W	Q	V	L	W	W	D	P	D	P	S	R
**VHv21**	Q	T	F	M	Y	Q	M	R	S	W	D	P	Y	P	S	R
**VHv22**	S	R	F	M	Y	Q	M	R	S	W	D	P	Y	P	Y	H
**VHv23**	Q	T	F	M	Y	Q	M	R	S	W	D	P	Y	P	Y	T
**VHv24**	S	R	F	R	W	Q	V	L	W	W	D	P	D	P	S	H

bioRxiv preprint doi: https://doi.org/10.1101/2025.10.22.684031; this version posted October 23, 2025. The copyright holder for this preprint (which was not certified by peer review) is the author/funder, who has granted bioRxiv a license to display the preprint in perpetuity. It is made available under aCC-BY 4.0 International license.

Supplementary Table 2. Most enriched combinatorial variants of N49P7-FR VL reformatted as IgG1 and evaluated for functionality and developability. For each variant, only the sampled amino acids are shown, ordered by position and numbered according to the Kabat scheme.

N49P7-FR variant	Amino acid position														
	**8**	**9**	**14**	**24**	**36**	**39**	**56**	**59**	**80**	**83**	**87**	**89**	**97**	**105**	**108**
**VL WT**	R	S	S	T	C	Q	S	P	D	D	F	W	A	T	G
**VLv1**	N	D	L	T	I	K	S	S	D	D	Y	F	A	W	N
**VLv2**	N	D	L	R	I	K	S	S	D	D	Y	F	A	W	N
**VLv3**	N	Y	L	T	L	Q	P	P	L	E	W	F	V	T	M
**VLv4**	N	H	S	T	L	K	P	S	L	D	W	W	V	T	M
**VLv5**	N	D	L	T	I	K	S	P	D	D	Y	F	A	W	N
**VLv6**	N	D	L	R	I	K	S	P	D	D	Y	F	A	W	N
**VLv7**	N	D	L	T	I	K	P	S	D	D	Y	F	A	W	N
**VLv8**	N	D	L	T	I	K	S	S	D	D	Y	F	A	W	G
**VLv9**	N	D	L	R	I	K	S	P	D	D	F	F	A	W	N
**VLv10**	N	D	L	T	I	K	S	S	L	D	W	W	V	T	M
**VLv11**	N	Y	L	T	I	K	S	S	D	D	Y	F	A	W	N
**VLv12**	N	H	S	T	I	K	S	S	D	D	Y	F	A	W	N
**VLv13**	N	H	S	T	L	K	S	S	D	D	Y	F	A	W	N
**VLv14**	N	D	L	T	I	K	S	S	D	D	Y	F	A	T	G
**VLv15**	R	H	L	T	I	K	S	S	D	D	Y	F	A	W	N
**VLv16**	N	H	L	T	I	K	S	S	D	D	Y	F	A	W	N
**VLv17**	N	D	L	T	I	K	P	S	L	D	W	W	V	T	M
**VLv18**	N	D	L	T	I	K	S	S	D	D	Y	F	A	W	T
**VLv19**	N	H	S	T	L	K	P	S	D	D	Y	F	A	W	N
**VLv20**	N	D	L	T	I	K	P	P	L	E	W	F	V	T	M
**VLv21**	R	S	L	T	I	K	S	S	D	D	Y	F	A	W	N

bioRxiv preprint doi: https://doi.org/10.1101/2025.10.22.684031; this version posted October 23, 2025. The copyright holder for this preprint (which was not certified by peer review) is the author/funder, who has granted bioRxiv a license to display the preprint in perpetuity. It is made available under aCC-BY 4.0 International license.

Supplementary Table 3. Most enriched combinatorial variants of N49P7-FR VHv1 reformatted as IgG1 and evaluated for functionality and developability. For each variant, only the sampled amino acids are shown, ordered by position and numbered according to the Kabat scheme.

N49P7-FR variant	Amino acid position																										
	3	6	10	11	12	25	39	40	43	45	48	51	53	54	57	66	67	69	75	76a	76c	76d	76f	93	98	100c	100e	100g
**VH WT**	D	Q	V	V	K	Q	R	A	Q	P	M	M	M	G	V	R	V	M	Q	P	D	P	W	V	N	R	E	S
**VHv1**	D	Q	V	V	K	Q	R	A	Q	P	M	M	M	Y	M	R	V	M	W	P	D	P	W	V	N	H	E	S
**VHv1-1**	D	Q	V	T	K	Q	Q	A	Q	P	M	I	Y	W	V	R	V	L	Q	P	D	P	R	V	N	L	D	D
**VHv1-2**	D	Q	V	T	K	S	Q	A	Q	P	M	I	Y	Y	V	R	L	L	Q	P	D	P	R	V	N	L	D	D
**VHv1-3**	D	Q	V	T	K	Q	Q	A	Q	P	M	I	Y	W	V	R	V	M	Q	P	D	P	R	V	N	L	E	D
**VHv1-4**	D	Q	V	T	K	S	Q	A	Q	P	L	I	Y	W	V	R	L	L	Q	P	D	P	W	V	N	L	E	D
**VHv1-5**	D	M	Q	V	A	Q	Q	A	Q	P	L	I	Y	W	V	R	M	L	Q	R	D	P	R	A	N	H	D	D
**VHv1-6**	D	Q	V	T	K	Q	Q	A	Q	P	L	I	Y	Y	I	R	V	L	Q	P	D	P	R	V	S	L	E	D
**VHv1-7**	D	Q	V	T	K	Q	Q	A	Q	P	M	I	Y	W	I	R	V	L	Q	P	D	P	R	V	N	L	D	S
**VHv1-8**	D	Q	V	V	K	S	Q	A	K	P	M	I	Y	W	V	R	V	L	Q	P	D	P	R	V	N	L	E	D
**VHv1-9**	D	Q	V	T	K	Q	Q	A	K	P	M	M	Y	W	V	R	L	L	Q	P	D	P	W	V	N	L	E	S
**VHv1-10**	D	Q	V	T	K	Q	Q	A	Q	P	M	M	Y	W	I	R	L	L	Q	P	D	P	R	V	N	L	D	S
**VHv1-11**	D	Q	V	V	K	Q	Q	A	Q	P	L	I	Y	W	V	R	V	L	Q	P	D	P	R	V	N	L	D	D
**VHv1-12**	D	Q	V	T	K	S	Q	A	Q	P	M	I	Y	W	V	R	V	L	Q	P	D	P	R	V	N	L	E	D
**VHv1-13**	D	Q	V	T	K	Q	Q	A	K	P	M	M	Y	W	I	R	V	L	Q	P	D	P	R	V	N	L	E	D
**VHv1-14**	D	Q	V	T	K	Q	Q	A	Q	P	M	I	Y	W	V	R	V	L	Q	P	D	P	R	V	N	L	D	S
**VHv1-15**	D	Q	V	T	K	Q	Q	A	Q	P	L	I	Y	W	V	R	V	L	Q	P	D	P	R	V	N	L	E	S
**VHv1-16**	D	Q	V	T	K	S	Q	A	K	P	M	I	Y	W	V	R	V	L	Q	P	D	P	R	V	N	L	E	D
**VHv1-17**	D	M	Q	T	A	S	Q	A	Q	P	M	I	Y	Y	V	R	V	M	Q	P	D	R	R	A	N	L	D	S
**VHv1-18**	D	Q	V	V	K	Q	Q	A	Q	P	M	M	Y	W	V	R	I	L	Q	P	D	P	R	V	N	L	D	D
**VHv1-19**	D	Q	V	T	A	S	Q	R	Q	P	L	I	Y	W	V	R	L	L	Q	P	D	P	R	A	N	H	D	D
**VHv1-20**	D	Q	V	T	K	Q	Q	A	Q	P	M	M	Y	Y	V	R	V	L	Q	P	D	R	R	V	N	L	D	D
**VHv1-21**	D	Q	V	T	K	Q	Q	A	Q	P	M	I	Y	Y	V	R	L	L	Q	R	D	P	R	V	N	L	D	D
**VHv1-22**	D	M	V	V	A	Q	Q	R	Q	P	M	I	Y	Y	V	R	M	L	Q	R	D	P	R	A	N	H	D	D
**VHv1-23**	D	Q	V	T	K	Q	Q	A	K	P	M	I	Y	Y	V	R	I	M	Q	P	D	P	W	V	S	L	E	D
**VHv1-24**	D	Q	V	T	K	Q	Q	A	Q	P	M	I	Y	W	V	R	V	L	Q	P	D	P	R	V	N	L	E	D
**VHv1-25**	D	M	V	V	A	S	Q	R	Q	P	M	M	Y	W	V	R	V	L	Q	P	D	P	W	A	N	L	D	S
**VHv1-26**	D	Q	V	T	K	Q	R	A	Q	P	M	I	Y	W	V	R	L	L	Q	P	D	P	W	V	N	L	D	S
**VHv1-27**	D	Q	V	T	K	S	Q	A	Q	P	L	M	Y	W	V	R	L	L	Q	P	D	R	R	V	N	H	E	D
**VHv1-28**	D	Q	V	T	K	Q	R	A	Q	P	M	I	Y	W	V	R	I	L	Q	P	D	P	R	V	N	L	D	D
**VHv1-29**	D	Q	V	T	K	Q	Q	A	Q	P	L	I	Y	W	V	R	V	L	Q	P	D	P	R	V	N	L	D	D
**VHv1-30**	D	Q	V	T	K	S	Q	A	K	P	L	I	Y	W	V	R	V	L	Q	P	D	P	R	V	N	L	E	D
**VHv1-31**	D	Q	V	V	K	S	R	A	Q	P	M	I	Y	W	V	R	L	L	Q	P	D	P	R	V	N	L	D	D
**VHv1-32**	D	Q	V	V	K	Q	Q	A	Q	P	M	I	Y	W	V	R	V	L	Q	P	D	R	R	V	N	L	E	D
**VHv1-33**	D	Q	Q	V	K	S	Q	R	Q	P	M	I	Y	W	V	R	V	L	Q	P	D	P	R	A	N	L	E	D
**VHv1-34**	D	Q	V	T	K	Q	Q	A	K	P	M	M	Y	W	V	R	V	L	Q	P	D	P	R	V	N	L	D	D
**VHv1-35**	D	Q	V	V	K	Q	Q	A	Q	P	L	I	Y	W	V	K	V	M	Q	P	D	P	R	V	N	L	D	D
**VHv1-36**	D	M	Q	V	A	S	Q	A	Q	P	M	I	Y	W	V	R	V	M	Q	P	D	R	R	A	N	L	D	D
**VHv1-37**	D	Q	V	T	K	S	Q	A	Q	P	M	M	Y	Y	I	R	V	M	Q	P	D	P	R	V	N	L	E	S
**VHv1-38**	D	Q	V	V	K	Q	Q	A	Q	P	M	M	Y	Y	V	R	I	M	Q	P	D	R	R	V	N	L	D	D
**VHv1-39**	D	M	Q	V	A	Q	Q	R	Q	P	M	I	Y	W	I	K	V	M	Q	P	D	P	R	V	N	L	D	D
**VHv1-40**	D	Q	V	T	K	Q	Q	A	K	P	M	I	Y	Y	V	R	I	M	Q	P	D	P	R	V	S	L	E	D
**VHv1-41**	D	Q	V	V	K	Q	R	A	Q	P	M	I	Y	W	V	R	L	L	Q	P	D	P	R	V	N	L	D	S
**VHv1-42**	D	Q	V	V	A	S	Q	A	Q	P	L	I	Y	W	V	K	V	L	Q	P	R	P	W	A	N	L	D	D
**VHv1-43**	D	Q	V	T	K	S	Q	A	Q	P	M	I	Y	W	I	R	I	L	Q	P	D	P	R	V	N	L	D	D
**VHv1-44**	D	Q	V	T	K	S	Q	A	Q	P	M	I	Y	W	V	R	V	L	Q	P	D	P	R	V	N	L	D	D
**VHv1-45**	D	Q	V	V	K	Q	Q	A	Q	P	M	M	Y	W	I	R	V	M	Q	P	D	P	R	V	N	L	D	D
**VHv1-46**	D	Q	V	T	K	S	Q	A	Q	P	L	M	Y	W	V	R	L	M	Q	P	D	P	R	V	N	L	D	D
**VHv1-47**	D	Q	V	V	K	Q	Q	A	Q	P	M	I	Y	W	V	R	V	L	Q	P	D	P	R	V	N	L	D	D
**VHv1-48**	D	Q	V	V	K	S	Q	A	K	P	M	M	Y	Y	I	R	V	L	Q	P	D	P	R	V	N	L	D	S
**VHv1-49**	D	Q	V	T	K	Q	Q	A	K	P	M	M	Y	Y	V	R	L	L	Q	R	D	P	R	V	N	H	D	D
**VHv1-50**	D	Q	V	T	K	Q	Q	A	Q	P	M	I	Y	Y	V	R	L	L	Q	P	D	P	R	V	N	L	D	D
**VHv1-51**	D	Q	V	V	K	Q	Q	A	Q	P	M	I	Y	W	V	R	L	L	Q	P	D	R	R	V	N	L	D	S
**VHv1-52**	D	Q	V	V	K	Q	Q	A	K	P	L	M	Y	W	V	R	M	L	Q	P	D	P	R	V	N	L	E	D
**VHv1-53**	D	Q	V	T	K	Q	Q	A	K	P	M	I	Y	W	L	R	L	L	Q	P	D	P	R	V	N	L	D	D
**VHv1-54**	D	Q	V	V	K	Q	Q	A	K	P	M	I	Y	W	I	R	M	L	Q	P	D	P	R	V	N	L	D	D
**VHv1-55**	D	Q	V	T	K	Q	Q	A	Q	P	M	I	Y	Y	I	R	L	L	Q	P	D	R	R	V	N	L	E	D
**VHv1-56**	D	Q	V	T	K	Q	Q	A	Q	P	M	I	Y	W	I	R	L	L	Q	P	D	P	W	V	N	L	D	D
**VHv1-57**	D	M	V	T	A	Q	Q	R	Q	P	L	I	Y	W	I	K	V	M	Q	P	D	P	W	A	N	L	E	S
**VHv1-58**	D	M	Q	V	A	Q	Q	R	Q	P	L	I	Y	Y	I	R	I	L	Q	R	D	P	R	A	N	H	E	D
**VHv1-59**	D	Q	V	T	K	Q	Q	A	Q	P	L	I	Y	W	V	R	V	L	Q	P	D	P	R	V	N	L	E	D
**VHv1-60**	D	Q	V	T	K	S	Q	A	Q	P	M	M	Y	W	I	R	V	M	Q	P	D	P	R	V	N	L	D	D
**VHv1-61**	D	Q	V	T	K	Q	Q	A	K	P	M	M	Y	Y	V	R	L	M	Q	P	D	P	R	V	N	L	D	S
**VHv1-62**	D	Q	V	V	A	Q	Q	A	Q	P	L	M	Y	W	V	R	V	M	Q	P	D	R	R	A	N	H	D	D
**VHv1-63**	D	Q	V	T	K	Q	Q	A	K	P	M	I	Y	W	V	R	V	M	Q	P	D	P	W	V	N	L	D	D
**VHv1-64**	D	Q	V	V	K	S	Q	A	Q	P	M	I	Y	W	V	R	V	L	Q	P	D	P	R	V	N	L	E	D
**VHv1-65**	D	Q	V	T	K	Q	Q	A	K	P	M	I	Y	Y	I	R	M	L	Q	P	D	P	R	V	S	L	D	D
**VHv1-66**	D	Q	V	V	K	Q	Q	A	Q	P	M	I	Y	W	V	R	L	L	Q	P	D	P	R	V	N	H	D	D
**VHv1-67**	D	Q	V	T	K	Q	Q	A	Q	F	M	M	Y	W	V	R	L	L	Q	P	D	P	W	V	N	L	D	S
**VHv1-68**	D	Q	V	T	K	Q	Q	A	Q	P	M	M	Y	Y	V	R	M	M	Q	P	D	R	R	V	N	L	D	D
**VHv1-69**	D	Q	V	T	K	Q	Q	A	Q	P	M	M	Y	W	V	R	V	L	Q	P	D	P	R	V	N	L	D	D
**VHv1-70**	D	M	V	V	A	S	Q	R	Q	P	M	M	Y	W	V	R	V	M	Q	P	D	P	W	A	N	L	D	D
**VHv1-71**	D	Q	V	V	K	Q	Q	A	Q	P	M	I	Y	W	V	R	V	L	Q	P	D	P	R	V	N	L	E	D
**VHv1-72**	D	Q	V	T	K	S	Q	A	K	P	M	M	Y	W	V	R	V	L	Q	P	D	P	W	V	N	H	D	S
**VHv1-73**	D	Q	V	V	K	Q	Q	A	Q	P	M	I	Y	W	V	R	M	M	Q	P	D	P	R	V	N	L	E	D
**VHv1-74**	D	Q	V	T	K	Q	Q	A	K	P	M	I	Y	Y	I	R	L	L	Q	R	D	P	R	V	N	H	D	D
**VHv1-75**	D	Q	V	T	K	Q	Q	A	Q	P	M	I	Y	Y	V	R	V	L	Q	P	D	R	R	V	N	L	D	D
**VHv1-76**	D	Q	V	V	K	S	Q	A	Q	P	M	I	Y	Y	V	R	L	L	Q	P	D	P	R	V	N	L	D	D
**VHv1-77**	D	Q	V	T	K	Q	Q	A	Q	P	L	I	Y	W	V	R	V	L	Q	P	D	P	R	V	N	L	D	S
**VHv1-78**	D	Q	V	V	K	S	Q	A	Q	P	M	I	Y	Y	I	R	L	L	Q	P	D	R	R	V	N	L	D	D
**VHv1-79**	D	Q	V	V	K	Q	Q	A	Q	P	L	M	Y	Y	V	R	L	L	Q	P	D	P	W	V	N	L	D	S
**VHv1-80**	D	Q	V	T	K	S	Q	A	K	P	M	M	Y	W	V	R	V	L	Q	P	D	P	R	V	N	L	D	D
**VHv1-81**	D	Q	V	T	K	Q	Q	A	Q	P	L	I	Y	W	V	R	M	L	Q	R	D	P	R	A	N	H	D	D
**VHv1-82**	D	M	V	T	A	S	Q	A	K	P	L	I	Y	W	I	K	V	L	Q	P	D	R	R	A	N	L	E	D
**VHv1-83**	D	Q	V	T	K	Q	Q	A	Q	P	M	I	Y	W	V	R	V	L	Q	P	D	P	R	V	N	H	D	D
**VHv1-84**	D	Q	V	T	K	S	Q	A	Q	P	M	I	Y	Y	V	R	L	L	Q	P	D	P	R	V	N	L	E	D
**VHv1-85**	D	M	V	T	A	Q	R	A	K	P	M	I	Y	Y	V	R	V	M	Q	P	D	P	W	A	S	L	D	S
**VHv1-86**	D	Q	V	T	K	S	Q	A	Q	P	M	M	Y	Y	V	R	V	L	Q	P	D	R	R	V	N	L	D	D
**VHv1-87**	D	Q	V	V	K	Q	Q	A	Q	P	M	I	Y	W	V	R	V	L	Q	P	D	P	R	V	N	L	D	S
**VHv1-88**	D	Q	V	V	K	Q	Q	A	Q	P	M	M	Y	Y	V	R	V	L	Q	P	D	R	R	V	N	L	D	D
**VHv1-89**	D	Q	V	V	K	Q	R	A	Q	P	L	I	Y	W	V	R	V	L	Q	P	D	P	R	V	N	L	D	D
**VHv1-90**	D	Q	V	V	K	S	Q	A	Q	P	L	M	Y	W	V	R	M	L	Q	P	D	P	R	V	N	L	D	D
**VHv1-91**	D	Q	V	V	K	S	Q	R	Q	P	L	M	Y	Y	V	R	L	L	Q	R	D	P	R	A	N	H	D	D
**VHv1-92**	K	M	Q	V	A	S	Q	R	Q	P	M	M	Y	W	I	K	V	M	Q	P	D	P	R	V	N	L	E	D
**VHv1-93**	D	M	V	V	K	S	Q	A	K	P	M	M	Y	W	V	R	V	L	Q	P	D	R	R	A	N	H	D	S
**VHv1-94**	D	Q	V	V	K	Q	Q	A	Q	P	M	I	Y	W	V	R	V	M	Q	P	D	P	R	V	N	L	D	D
**VHv1-95**	K	M	Q	T	A	S	Q	A	Q	P	L	I	Y	Y	I	R	L	L	Q	P	D	R	R	A	N	H	D	D
**VHv1-96**	D	M	Q	V	A	Q	Q	A	K	P	M	M	Y	W	V	R	L	L	Q	P	R	P	R	A	N	H	D	D

bioRxiv preprint doi: https://doi.org/10.1101/2025.10.22.684031; this version posted October 23, 2025. The copyright holder for this preprint (which was not certified by peer review) is the author/funder, who has granted bioRxiv a license to display the preprint in perpetuity. It is made available under aCC-BY 4.0 International license.

Supplementary Table 4. Developability characteristics of the most enriched combinatorial variants of N49P7-FR VH reformatted as IgG1. Red shading indicates developability liabilities, with color intensity proportional to severity. Variants in bold demonstrated the most favorable biophysical profiles and were selected for HIV neutralization analysis (Supplementary Fig. 14).

Antibody/ N49P7-FR VH variant	Purification yield[1] [mg/L]	Precipitation[2]	Polyreactivity with CHO-SMP[3] at 50 µg/mL Ab [A_{405}]	SEC t_R[4] [min]
Adalimumab[5]	74	0	0.21	4.1
4E10[6]	-	-	3.58	-
N49P7	61	0	0.17	4.3
N49P7-FR	71	0	0.24	4.1
**VHv1**	9	++	0.83	5.5
VHv2	28	++	1.62	-
VHv3	28	++	1.76	-
**VHv4**	67	++	0.71	4.5
VHv5	17	++	0.71	0.3
VHv6	12	++	1.6	-
VHv7	24	++	3.19	-
VHv8	20	+	1.11	-
VHv9	28	+	0.91	0.4
**VHv10**	43	+	0.29	4.6
VHv11	14	++	3.06	-
**VHv13**	33	++	0.9	5.5
VHv14	27	++	1.4	-
VHv15	31	++	3.16	-
VHv16	35	+	1.77	-
VHv17	17	++	2	-
VHv18	21	+	1.44	-
VHv19	36	++	1.51	-
VHv20	31	+	1.31	-
VHv21	41	++	1.57	-
VHv22	7	++	2.33	-
VHv23	21	++	0.98	-
VHv24	15	+	1.72	-
**VHv1+Y54G**	18	++	0.55	4.8

[1] Antibody yield [mg] after purification from 1 L culture of Expi293F cells.
[2] Precipitation after antibody elution, low pH hold (1 hour at pH 3.0), and buffer exchange to PBS; no (0), low (+), high (++) precipitation.
[3] CHO-SMP (Chinese hamster ovary soluble membrane proteins) used as a polyspecificity reagent in ELISA.
[4] SEC retention time (t_R) measured on the 15-cm column TSKgel SuperSW mAb HR (Tosoh Bioscience) for the VH variants with the lowest polyreactivity (A_{405}<1.0).
[5] FDA-approved reference antibody.
[6] Polyreactive antibody used as control in PSR-ELISA.

bioRxiv preprint doi: https://doi.org/10.1101/2025.10.22.684031; this version posted October 23, 2025. The copyright holder for this preprint (which was not certified by peer review) is the author/funder, who has granted bioRxiv a license to display the preprint in perpetuity. It is made available under aCC-BY 4.0 International license.

Supplementary Table 5. Developability characteristics of the most enriched combinatorial variants of N49P7-FR VL reformatted as IgG1. Red shading indicates developability liabilities, with color intensity proportional to severity. Variants in bold demonstrated the most favorable biophysical profiles and were selected for HIV neutralization analysis (Supplementary Fig. 15).

Antibody/ N49P7-FR VH/VL variant	Purification yield[1] [mg/L]	Precipitation[2]	Polyreactivity with PSR[3] at 50 μg/mL Ab [A_{405}]			SEC t_R[4] [min]	T_m[5] [°C]
			CHO-SMP	Insulin	ssDNA		
Adalimumab[6]	66	0	0.10	0.07	0.10	8.3	71.0
Ipilimumab[7]	-	-	-	-	-	9.5	-
4E10[8]	-	-	2.52	2.73	2.77	-	-
N49P7-FR	89	0	0.27	1.26	0.74	8.4	66.5
VHv10/VL-WT	37	++	0.35	1.08	1.72	9.6	67.0
VHv10/VLv1	98	0	0.17	0.33	0.18	9.3	69.5
VHv10/VLv2	80	0	0.22	0.48	0.21	9.5	69.0
**VHv10/VLv3**	97	0	0.10	0.14	0.11	9.3	74.5
VHv10/VLv4	17	++	0.25	0.70	0.53	5.7; 9.4	67.5
VHv10/VLv5	99	0	0.14	0.35	0.15	9.3	70.0
VHv10/VLv6	73	0	0.17	0.43	0.16	9.5	69.5
VHv10/VLv7	108	0	0.16	0.29	0.15	9.3	71.0
VHv10/VLv8	67	++	0.19	0.44	0.16	9.3	69.0
**VHv10/VLv9**	77	0	0.14	0.27	0.15	9.5	74.5
VHv10/VLv10	28	+	0.21	0.55	0.26	5.7; 9.3	66.5
VHv10/VLv11	99	0	0.16	0.31	0.13	9.3	70.0
VHv10/VLv12	85	0	0.18	0.46	0.15	9.3	70.5
VHv10/VLv13	66	++	0.18	0.45	0.15	9.3	70.0
VHv10/VLv14	77	++	0.21	0.44	0.17	5.7; 9.2	67.0
VHv10/VLv15	89	+	0.19	0.41	0.17	9.4	71.5
VHv10/VLv16	100	0	0.14	0.31	0.15	9.4	70.5
VHv10/VLv17	77	++	0.16	0.37	0.21	5.7; 9.3	67.0
VHv10/VLv18	104	0	0.10	0.17	0.12	9.3	70.0
VHv10/VLv19	81	++	0.16	0.34	0.15	9.4	72.0
**VHv10/VLv20**	128	0	0.10	0.11	0.11	9.3	75.5
VHv10/VLv21	103	0	0.18	0.27	0.15	9.4	70.5

[1] Antibody yield [mg] after purification from 1 L culture of Expi293F cells.
[2] Precipitation after antibody elution, low pH hold (1 hour at pH 3.0), and buffer exchange to PBS; no (0), low (+), high (++) precipitation.
[3] Polyspecificity reagents (PSR) used in ELISA: CHO-SMP (Chinese hamster ovary soluble membrane proteins), insulin, and ssDNA.
[4] SEC retention time (t_R) measured on the 30-cm column TSKgel SuperSW mAb HR (Tosoh Bioscience).
[5] Melting temperature (T_m) measured by DSF.
[6] FDA-approved reference antibody.
[7] FDA-approved antibody used as control in SEC.
[8] Polyreactive antibody used as control in PSR-ELISA.

bioRxiv preprint doi: https://doi.org/10.1101/2025.10.22.684031; this version posted October 23, 2025. The copyright holder for this preprint (which was not certified by peer review) is the author/funder, who has granted bioRxiv a license to display the preprint in perpetuity. It is made available under aCC-BY 4.0 International license.

Supplementary Table 6. Developability characteristics of top N49P7-FR VH variants shuffled with VLv3. Red shading indicates developability liabilities, with color intensity proportional to severity. Variants were subsequently evaluated for HIV neutralization (Supplementary Fig. 16).

Antibody/ N49P7-FR VH/VL variant	Purification yield[1] [mg/L]	Precipitation[2]	Polyreactivity with PSR[3] at 50 µg/mL Ab [A_{405}]			SEC t_R[4] [min]	T_m[5] [°C]
			CHO-SMP	Insulin	ssDNA		
Adalimumab[6]	76	0	0.10	0.08	0.07	8.3	71.0
Ipilimumab[7]	-	-	-	-	-	9.5	-
4E10[8]	-	-	3.05	3.53	2.43	-	-
N49P7	72	0	0.63	2.45	2.82	9.0	65.0
N49P7-FR	70	0	0.23	1.44	0.42	8.4	66.5
VHv1/VL-WT	11	++	0.99	2.25	3.17	11.9	68.0
VHv1/VLv3	16	+	0.19	0.59	0.21	11.7	69.5
VHv10/VL-WT	32	++	0.25	1.01	0.68	9.6	66.0
VHv10/VLv3	112	0	0.10	0.12	0.08	9.3	74.5
VHv13/VL-WT	29	++	1.32	2.92	2.92	11.7	68.0
VHv13/VLv3	98	+	0.16	0.32	0.11	12.1	73.0

[1] Antibody yield [mg] after purification from 1 L culture of Expi293F cells.
[2] Precipitation after antibody elution, low pH hold (1 hour at pH 3.0), and buffer exchange to PBS; no (0), low (+), high (++) precipitation.
[3] Polyspecificity reagents (PSR) used in ELISA: CHO-SMP (Chinese hamster ovary soluble membrane proteins), insulin, and ssDNA.
[4] SEC retention time (t_R) measured on the 30-cm column TSKgel SuperSW mAb HR (Tosoh Bioscience).
[5] Melting temperature (T_m) measured by DSF.
[6] FDA-approved reference antibody.
[7] FDA-approved antibody used as control in SEC.
[8] Polyreactive antibody used as control in PSR-ELISA.

bioRxiv preprint doi: https://doi.org/10.1101/2025.10.22.684031; this version posted October 23, 2025. The copyright holder for this preprint (which was not certified by peer review) is the author/funder, who has granted bioRxiv a license to display the preprint in perpetuity. It is made available under aCC-BY 4.0 International license.

Supplementary Table 7. Developability characteristics of the most enriched combinatorial variants of N49P7-FR VHv1 paired with VLv3 and reformatted as IgG1. Red shading indicates developability liabilities, with color intensity proportional to severity. Variants in bold had the most favorable SEC profiles and were selected for HIV neutralization analysis (Supplementary Fig. 17).

Antibody/ N49P7-FR VH/VL variant	Polyreactivity with CHO-SMP[1] at 50 µg/mL Ab [A_{405}]	SEC t_R[2] [min]
Adalimumab[3]	0.07	8.3
4E10[4]	2.24	-
Golimumab[5]	-	9.1
Ipilimumab[5]	-	9.5
N49P7-FR	0.12	8.4
VHv1/VLv3	0.14	11.7
VHv1-1/VLv3	0.14	11.9
**VHv1-2/VLv3**	0.13	9.5
VHv1-3/VLv3	0.16	11.9
VHv1-4/VLv3	0.14	11.5
VHv1-5/VLv3	0.22	12.1
**VHv1-6/VLv3**	0.15	9.3
VHv1-7/VLv3	0.18	12.8
VHv1-8/VLv3	0.17	12.3
VHv1-9/VLv3	0.17	12.9
VHv1-10/VLv3	0.20	13.5
VHv1-11/VLv3	0.19	11.9
VHv1-12/VLv3	0.17	12.2
VHv1-13/VLv3	0.18	12.2
VHv1-14/VLv3	0.21	13.0
VHv1-15/VLv3	0.17	12.9
VHv1-16/VLv3	0.16	12.3
VHv1-17/VLv3	0.17	10.2
VHv1-18/VLv3	0.17	12.2
VHv1-19/VLv3	0.14	12.0
VHv1-20/VLv3	0.25	5.2
VHv1-21/VLv3	0.22	9.9
VHv1-22/VLv3	0.16	9.8
**VHv1-23/VLv3**	0.15	9.2
VHv1-24/VLv3	0.16	11.8
VHv1-25/VLv3	0.13	12.4
VHv1-26/VLv3	0.15	12.5
VHv1-27/VLv3	0.33	13.1
VHv1-28/VLv3	0.14	12.0
VHv1-29/VLv3	0.13	11.9
VHv1-30/VLv3	0.12	12.3
VHv1-31/VLv3	0.14	12.5
VHv1-32/VLv3	0.27	12.5
VHv1-33/VLv3	0.15	12.2
VHv1-34/VLv3	0.15	12.5
VHv1-35/VLv3	0.14	5.2
VHv1-36/VLv3	0.33	12.9
VHv1-37/VLv3	0.15	10.1
VHv1-38/VLv3	0.36	10.0
VHv1-39/VLv3	0.17	11.9
**VHv1-40/VLv3**	0.16	9.5
VHv1-41/VLv3	0.18	13.4
VHv1-42/VLv3	0.28	13.1
VHv1-43/VLv3	0.17	12.2
VHv1-44/VLv3	0.18	12.3
VHv1-45/VLv3	0.15	12.3
VHv1-46/VLv3	0.20	12.9
VHv1-47/VLv3	0.13	12.0
VHv1-48/VLv3	0.16	10.3
VHv1-49/VLv3	0.20	10.1
**VHv1-50/VLv3**	0.12	9.5
VHv1-51/VLv3	0.34	14.7
VHv1-52/VLv3	0.13	12.2
VHv1-53/VLv3	0.15	12.1
VHv1-54/VLv3	0.14	11.9
VHv1-55/VLv3	0.16	9.9
VHv1-56/VLv3	0.14	11.3
VHv1-57/VLv3	0.11	12.2
**VHv1-58/VLv3**	0.13	9.7
VHv1-59/VLv3	0.12	11.8
VHv1-60/VLv3	0.13	12.8
VHv1-61/VLv3	0.16	10.2
VHv1-62/VLv3	0.42	13.0
VHv1-63/VLv3	0.14	11.6
VHv1-64/VLv3	0.15	12.3
**VHv1-65/VLv3**	0.16	9.5
VHv1-66/VLv3	0.15	11.9
VHv1-67/VLv3	0.14	12.6
VHv1-68/VLv3	0.25	10.0
VHv1-69/VLv3	0.16	12.3
VHv1-70/VLv3	0.14	11.5
VHv1-71/VLv3	0.12	11.8
VHv1-72/VLv3	0.13	13.1
VHv1-73/VLv3	0.12	11.8
VHv1-74/VLv3	0.17	10.1
VHv1-75/VLv3	0.19	9.9
**VHv1-76/VLv3**	0.10	9.6
VHv1-77/VLv3	0.11	13.2
VHv1-78/VLv3	0.24	10.1
VHv1-79/VLv3	0.11	9.8
VHv1-80/VLv3	0.15	13.2
VHv1-81/VLv3	0.28	12.9
VHv1-82/VLv3	0.24	13.2
VHv1-83/VLv3	0.11	11.9
**VHv1-84/VLv3**	0.10	9.5
**VHv1-85/VLv3**	0.12	9.5
VHv1-86/VLv3	0.28	10.2
VHv1-87/VLv3	0.15	13.2
VHv1-88/VLv3	0.30	5.2
VHv1-89/VLv3	0.14	12.2
VHv1-90/VLv3	0.19	12.8
VHv1-91/VLv3	0.21	10.2
VHv1-92/VLv3	0.27	13.2
VHv1-93/VLv3	0.89	16.0
VHv1-94/VLv3	0.15	12.2
VHv1-95/VLv3	0.26	10.5
VHv1-96/VLv3	0.26	13.5

[1] CHO-SMP (Chinese hamster ovary soluble membrane proteins) used as a polyspecificity reagent in ELISA.
[2] SEC retention time (t_R) measured on the 30-cm column TSKgel SuperSW mAb HR (Tosoh Bioscience).
[3] FDA-approved reference antibody.
[4] Polyreactive antibody used as control in PSR-ELISA.
[5] FDA-approved antibodies used as controls in SEC.

bioRxiv preprint doi: https://doi.org/10.1101/2025.10.22.684031; this version posted October 23, 2025. The copyright holder for this preprint (which was not certified by peer review) is the author/funder, who has granted bioRxiv a license to display the preprint in perpetuity. It is made available under aCC-BY 4.0 International license.

Supplementary Table 8. Developability characteristics of the most potent N49P7-FR VHv1 variants. Red shading indicates developability liabilities, with color intensity proportional to severity. Variants were subsequently evaluated for HIV neutralization (Supplementary Figs. 18–19).

Antibody/ N49P7-FR VH/VL variant	Purification yield[1] [mg/L]	Precipitation[2]	Polyreactivity with PSR[3] at 50 µg/mL Ab [A_{405}]			SEC t_R[4] [min]	T_m[5] [°C]
			CHO-SMP	Insulin	ssDNA		
Adalimumab[6]	71	0	0.07	0.07	0.08	8.3	71.0
4E10[7]	-	-	2.79	3.00	2.42	-	-
Golimumab[8]	-	-	-	-	-	9.1	-
Ipilimumab[8]	-	-	-	-	-	9.5	-
N49P7-FR	78	0	0.11	0.64	0.15	8.4	66.5
VHv1/VLv3	17	+	0.16	0.30	0.25	11.7	69.5
VHv1-23/VLv3	87	0	0.10	0.11	0.13	9.2	69.0
VHv1-65/VLv3	115	+	0.27	0.77	0.56	9.5	71.0
VHv1-85/VLv3	42	+	0.18	0.56	0.34	9.5	78.5

[1] Antibody yield [mg] after purification from 1 L culture of Expi293F cells.
[2] Precipitation after antibody elution, low pH hold (1 hour at pH 3.0), and buffer exchange to PBS; no (0), low (+) precipitation.
[3] Polyspecificity reagents (PSR) used in ELISA: CHO-SMP (Chinese hamster ovary soluble membrane proteins), insulin, and ssDNA.
[4] SEC retention time (t_R) measured on the 30-cm column TSKgel SuperSW mAb HR (Tosoh Bioscience).
[5] Melting temperature (T_m) measured by DSF.
[6] FDA-approved reference antibody.
[7] Polyreactive antibody used as control in PSR-ELISA.
[8] FDA-approved antibodies used as controls in SEC.

bioRxiv preprint doi: https://doi.org/10.1101/2025.10.22.684031; this version posted October 23, 2025. The copyright holder for this preprint (which was not certified by peer review) is the author/funder, who has granted bioRxiv a license to display the preprint in perpetuity. It is made available under aCC-BY 4.0 International license.

Supplementary Table 9. Kinetic parameters for N49P7 variants binding to a panel of HIV cross-clade gp120s. The association rate constant (k_a), dissociation rate constant (k_d), and equilibrium dissociation constant (K_D) were derived from SPR sensorgrams globally fit to either a 1:1 Langmuir binding model (one k_a, k_d, and K_D value) or a heterogeneous ligand model (two sets of k_a, k_d, and K_D values). eN49P7-FRv1-23 exhibited improved binding kinetics relative to the parental N49P7-FR for 9 of the 11 tested gp120s (92BR020, 25710, IAVI-C22, BJOX2000, CH119, 6041.v3, CAP45, TRO.11, and Ce1176).

Antigen (gp120)	Antibody (IgG)	k_a1 [1/Ms]	k_d1 [1/s]	K_D1 [M]	k_a2 [1/Ms]	k_d2 [1/s]	K_D2 [M]
92BR020 (clade B)	N49P7	1.5×10^5	1.4×10^{-2}	9.3×10^{-8}	2.0×10^4	6.1×10^{-4}	3.1×10^{-8}
	N49P7-FR	6.7×10^3	5.7×10^{-4}	8.5×10^{-8}	6.1×10^3	7.8×10^{-3}	$>1.0 \times 10^{-6}$
	eN49P7-FRv1-23	2.0×10^5	1.1×10^{-2}	5.2×10^{-8}	8.9×10^4	7.9×10^{-4}	8.8×10^{-9}
25710 (clade C)	N49P7	9.6×10^4	1.6×10^{-2}	1.6×10^{-7}	2.1×10^4	7.6×10^{-4}	3.6×10^{-8}
	N49P7-FR	3.6×10^4	1.5×10^{-2}	4.2×10^{-7}	1.1×10^4	9.4×10^{-4}	8.9×10^{-8}
	eN49P7-FRv1-23	4.0×10^5	1.4×10^{-3}	3.0×10^{-9}	3.4×10^4	1.8×10^{-5}	5.4×10^{-10}
IAVI-C22 (clade C)	N49P7	4.9×10^3	1.1×10^{-3}	2.3×10^{-7}			
	N49P7-FR	2.0×10^2	1.4×10^{-3}	$>1.0 \times 10^{-6}$			
	eN49P7-FRv1-23	1.4×10^4	9.2×10^{-4}	6.5×10^{-8}			
BJOX2000 (clade BC)	N49P7	6.8×10^2	2.4×10^{-3}	$>1.0 \times 10^{-6}$			
	N49P7-FR	2.8×10^2	2.5×10^{-3}	$>1.0 \times 10^{-6}$			
	eN49P7-FRv1-23	1.8×10^4	9.2×10^{-4}	5.0×10^{-8}			
93TH057 (clade AE)	N49P7	2.2×10^4	8.5×10^{-4}	3.8×10^{-8}			
	N49P7-FR	1.2×10^5	2.2×10^{-3}	1.9×10^{-8}			
	eN49P7-FRv1-23	5.1×10^4	1.5×10^{-3}	2.9×10^{-8}			
CH119 (clade BC)	N49P7	1.1×10^4	7.4×10^{-4}	6.5×10^{-8}			
	N49P7-FR	7.4×10^3	8.0×10^{-4}	1.1×10^{-7}			
	eN49P7-FRv1-23	2.7×10^4	5.3×10^{-4}	2.0×10^{-8}			
6041.v3 (clade AC)	N49P7	3.1×10^4	3.2×10^{-3}	1.0×10^{-7}			
	N49P7-FR	1.4×10^4	2.6×10^{-3}	1.9×10^{-7}			
	eN49P7-FRv1-23	4.4×10^4	1.0×10^{-3}	2.4×10^{-8}			
CAP45 (clade C)	N49P7	1.4×10^4	1.7×10^{-3}	1.2×10^{-7}			
	N49P7-FR	1.2×10^4	1.7×10^{-3}	1.4×10^{-7}			
	eN49P7-FRv1-23	1.0×10^5	3.1×10^{-3}	3.1×10^{-8}			
TRO.11 (clade B)	N49P7	4.5×10^3	1.1×10^{-3}	2.5×10^{-7}			
	N49P7-FR	9.9×10^3	1.2×10^{-3}	1.2×10^{-7}			
	eN49P7-FRv1-23	1.7×10^4	7.4×10^{-4}	4.4×10^{-8}			
Ce1176 (clade C)	N49P7	1.5×10^4	5.5×10^{-4}	3.6×10^{-8}			
	N49P7-FR	9.4×10^3	5.9×10^{-4}	6.3×10^{-8}			
	eN49P7-FRv1-23	4.2×10^4	6.9×10^{-4}	1.7×10^{-8}			
CNE8 (clade AE)	N49P7	1.1×10^4	7.9×10^{-4}	7.3×10^{-8}			
	N49P7-FR	8.8×10^4	1.3×10^{-3}	1.5×10^{-8}			
	eN49P7-FRv1-23	2.4×10^4	1.1×10^{-3}	4.7×10^{-8}			

bioRxiv preprint doi: https://doi.org/10.1101/2025.10.22.684031; this version posted October 23, 2025. The copyright holder for this preprint (which was not certified by peer review) is the author/funder, who has granted bioRxiv a license to display the preprint in perpetuity. It is made available under aCC-BY 4.0 International license.

Supplementary Table 10. Cryo-EM data collection, refinement, and validation statistics.

	N49P7-FR Fab + BG505 MD39 SOSIP + RM20A3 Fab (EMD-71307) (PDB 9P6E)	eN49P7-FRv1-23 Fab + BG505 MD39 SOSIP + RM20A3 Fab (EMD-71308) (PDB 9P6G)
**Data collection and processing**		
Microscope	TFS Glacios	TFS Glacios
Voltage (keV)	200	200
Camera	TFS Falcon 4i	TFS Falcon 4i
Collection mode	Counting	Counting
Magnification	190,000x	190,000x
Pixel size at detector (Å)	0.718	0.718
Total electron exposure (e–/Å^2)	45	45
Exposure rate (e-/pixel/sec)	9.05	9.05
Number of EER frames	40	40
Defocus range (μm)	-0.8 to -1.8	-0.7 to -1.8
Automation software	EPU	EPU
Micrographs collected (no.)	4,683	8,003
Micrographs used (no.)	3,881	7,598
Initial particle images (no.)	465,429	913,773
Final particle images (no.)	146,996	212,503
Symmetry	C3	C3
Map resolution (masked/unmasked Å)	2.7/3.1	2.6/3.1
FSC threshold	0.143	0.143
Map sharpening *B* factor (Å^2)	-75.9	-77.8
Map resolution range (Å)	2.3-3.5	2.3-3.5
**Refinement**		
Initial model used (PDB code)	6DFG, 6BCK	6DFG, 6BCK
Refinement package	Phenix real space refine	Phenix real space refine
Model resolution (Å)	2.8	2.7
FSC threshold	0.5	0.5
EMRinger score	5.04	4.36
CC (mask)	0.84	0.84
Model composition		
Non-hydrogen atoms	24,678	24,684
Protein residues	3,054	3,039
Ligands	69	72
Mean B factors (Å^2)		
Protein	41.96	25.80
Ligand	65.12	43.86
R.m.s. deviations		
Bond lengths (Å)	0.005	0.005
Bond angles (°)	0.932	0.860
Validation		
MolProbity score	0.89	0.99
Clashscore	0.91	1.28
Poor rotamers (%)	0.92	0.46
Ramachandran plot		
Favored (%)	97.39	97.28
Allowed (%)	2.51	2.72
Disallowed (%)	0.10	0.00
Cβ outliers (%)	0.00	0.00
CaBLAM outliers (%)	1.23	1.86

bioRxiv preprint doi: https://doi.org/10.1101/2025.10.22.684031; this version posted October 23, 2025. The copyright holder for this preprint (which was not certified by peer review) is the author/funder, who has granted bioRxiv a license to display the preprint in perpetuity. It is made available under aCC-BY 4.0 International license.

Supplementary Table 11. Amino acid variation at N49P7-FR and eN49P7-FRv1-23 contact sites in the HIV Env of 206 strains. Contact sites were determined by cryo-EM analysis of N49P7-FR or eN49P7-FRv1-23 in complex with BG505 MD39 SOSIP. For each strain, amino acid substitutions relative to BG505 MD39 SOSIP at these positions are shown; gray shading indicates no substitution. All Env sequences were derived from pseudoviruses used in the *in vitro* neutralization assay, for which the potency of both bnAbs is reported as IC_{80} values. Strains highlighted in red have no mutations at any of the identified bnAb contact positions.

#	HIV strain	Clade	IC_{80}		Amino acid in the HIV Env gp120 (BG505) position that contacts N49P7-FR (□) and/or eN49P7-FRv1-23 VH (■)																	
			N49 P7-FR	eN49 P7-FR	P118 ■	K207 □	N279 □ ■	N280 □	A281 ■	K282 □ ■	Y318 □	S365 □ ■	D368 □ ■	M426 ■	Q428 ■	T455 ■	R456 □ ■	D457 □ ■	G458 □ ■	P470 ■	G472 □	D474 ■
1	H704_0855_080_EsN	B	0.004	0.002			D									M						
2	H704_0944_180_RE_cs	B	0.007	0.005								T		L								E
3	H703_1383_240_RE_pblib003_s	C	0.009	0.005					V							V						
4	Q842.d12	A	0.010	0.017								T										
5	H704_1183_220EsN	B	0.010	0.009			D															
6	V703_1383_240_RE_pblib002_s	C	0.012	0.007					V							V						
7	HIV-001428-2.42	C	0.012	0.006			D		V							V						
8	CNE52	BC	0.012	0.018												V						
9	6545.v4.c1	AC	0.015	0.032				S	V								S		Y			
10	R2184.c04	AE	0.015	0.028			D		T													N
11	H703_1383_240_RE_e5D3s	C	0.015	0.007					V							V						
12	H703_0636_200Es	C	0.016	0.014			D									V						
13	H704_1783_150_RE_cs	B	0.016	0.007			D			R	F											N
14	3301.v1.c24	AC	0.017	0.028			D		V													
15	H704_2684_181_RE_pblib006_s	B	0.018	0.007			D		V													N
16	H704_1535_030sN	B	0.023	0.005			D		T		F			L								N
17	H704_2684_181_RE_p001s	B	0.025	0.010			D		V													N
18	H703_1945_090s_3D5	C	0.026	0.026								A										
19	H704_0907_130sN	B	0.026	0.012			D		T			P										
20	89-F1_2_25	CD	0.027	>50					V			P					S		N			N
21	235-47	AG	0.027	0.019						G												N
22	H704_2095_130_RE_cs	B	0.028	0.015			D		T			A										
23	V703_3000_090_RE_pblib002_s	C	0.029	0.008			D		T													E
24	H704_2448_240_RE_cs	B	0.029	0.016										L								
25	V704_0372_250_RE_pblib002_s	B	0.031	0.007			D		S			A										N
26	H703_1828_220Es	C	0.033	0.009			D		V													
27	H703_1945_090s_2A3	C	0.035	0.007					V			A										
28	H703_1945_090s_2F1	C	0.035	0.025								A										
29	ZM109F.PB4	C	0.036	0.036			D															N
30	3415.v1.c1	A	0.036	0.082			D															
31	H703_0597_190_RE_e5A1s	C	0.041	0.049			D		V					L		V						
32	H704_1930_170_RE_c01s_1523A	B	0.042	0.010			D		T													N
33	3365.v2.c2	A	0.043	0.026																		
34	Q23.17	A	0.046	0.029																		
35	H704_1528_240_RE_pblib_001_s	B	0.046	0.016			D							I								N
36	263-8	AG	0.047	0.038																		
37	CNE58	BC	0.047	0.030					V							V						
38	Q769.d22	A	0.048	0.038								L										
39	H703_0967_040s	C	0.049	0.006			D		V													N
40	Du156.12	C	0.050	0.026			D		I													N
41	Q259.d2.17	A	0.050	0.045					G			A										
42	V703_0597_190_RE_pblib002_s	C	0.050	0.060			D		V					L		V						
43	V704_0372_250_RE_pblib001_s	B	0.051	0.010			D		S			A										N
44	ZM249M.PL1	C	0.052	0.050			D		V													N
45	C3347.c11	AE	0.053	0.057								L										N
46	X1193_c1	G	0.056	0.049			D									V						
47	191084 B7-19	A	0.057	0.052			D				F			V		V						N
48	V703_0712_250_RE_pblib002_s	C	0.057	0.007			D		T													
49	H703_0795_040s	C	0.058	0.054			D									V						N
50	BF1266.431a	C	0.060	0.026			D															
51	V703_1407_090_RE_pblib003_s	C	0.060	0.019												V						N
52	BJOX028000.10.3	AE	0.065	0.031																		N
53	WEAU_d15_410_787	B	0.066	0.026			D							R								N
54	V703_0597_190_RE_pblib003_s	C	0.066	0.066			D		V					L		V						
55	H703_1758_260_RE_cs	C	0.067	0.025			D					T										
56	H704_2065_060_RE_p001s	B	0.067	0.047																		N
57	X2088_c9	G	0.068	0.148								A										
58	H704_0847_030_EsN_01T	B	0.072	0.048						R		R										N
59	H704_1930_170_RE_c02s_1523G	B	0.072	0.015			D		T													N
60	H704_2555_240_RE_con_s_vpuG	B	0.073	0.022						Q												
61	V703_2372_170_RE_con_s	C	0.074	0.012			D		T							V						N
62	249M B10	C	0.074	0.097			D		V													N
63	R1166.c01	AE	0.074	0.062										L								N
64	H703_1783_170Es	C	0.074	0.012			D									V						
65	H703_1407_090s_4G4	C	0.075	0.029												V						N
66	1012_11_TC21_3257	B	0.076	0.064			D															

bioRxiv preprint doi: https://doi.org/10.1101/2025.10.22.684031; this version posted October 23, 2025. The copyright holder for this preprint (which was not certified by peer review) is the author/funder, who has granted bioRxiv a license to display the preprint in perpetuity. It is made available under aCC-BY 4.0 International license.

Supplementary Table 11. (continued)

#	HIV strain	Clade	IC$_{80}$		Amino acid in the HIV Env gp120 (BG505) position that contacts N49P7-FR (□) and/or eN49P7-FRv1-23 VH (■)																	
			N49 P7-FR	eN49 P7-FR	P118 ■	K207 □	N279 □ ■	N280 □	A281 ■	K282 □ ■	Y318 □	S365 □ ■	D368 □ ■	M426 ■	Q428 ■	T455 ■	R456 □ ■	D457 □ ■	G458 □ ■	P470 ■	G472 □	D474 ■
67	WITO4160.33	B	0.078	0.028					G													N
68	0815.v3.c3	ACD	0.078	0.021					G			A										
69	Ce0682_E4	C	0.079	0.019					V			A										
70	REJO4541.67	B	0.079	0.044			D															
71	TRO.11	B	0.080	0.046										L								N
72	HIV-16055-2.3	C	0.082	0.046					V			A				V						
73	Ce0393_C3	C	0.084	0.174																		
74	H704_1775_030cN_SynGtoA_567	B	0.086	0.022			D		T								K					N
75	CNE8	AE	0.087	0.030			D		V													N
76	H704_2788_060eN_12	B	0.089	0.016			D															N
77	RHPA4259.7	B	0.092	0.036					V													N
78	MS208.A1	A	0.094	0.055																		
79	H704_0496_040sN	B	0.099	0.018			D															
80	T255-34	AG	0.104	0.037																		
81	P0402_c2_11	G	0.106	0.086								A										
82	CNE19	BC	0.108	0.010					V							V						
83	211-9	AG	0.108	0.287																		
84	9004SS_A3_4	A	0.111	0.078								T										
85	ZM247v1(Rev-)	C	0.115	0.066					I													
86	Q461.e2	A	0.117	0.128																		
87	H704_2788_060eN_04	B	0.121	0.019			D															N
88	3016.v5.c45	D	0.122	0.077						Y		A										N
89	H703_0322_130s_M1I	C	0.128	0.043			D															
90	PVO.4	B	0.130	0.096																		
91	H704_3008_040EsN	B	0.134	0.042			D			R												
92	1006_11_C3_1601	B	0.137	0.053																		
93	BJOX025000.01.1	AE	0.138	0.141																		N
94	928-28	AG	0.141	0.173																		
95	231966.c02	D	0.145	0.058												A						
96	H704_0644_060sN_prelimSeq	B	0.148	0.032			D															N
97	H704_0847_030_EsN_02C	B	0.151	0.076						R		R										N
98	H703_1453_240_RE_e9B7s	C	0.153	0.031			D									V						
99	CNE53	BC	0.160	0.057												V						
100	BJOX015000.11.5	AE	0.163	0.063					V													N
101	V703_0712_250_RE_pblib001_s	C	0.168	0.020			D															
102	H704_0856_240_RE_pb001_s	B	0.168	0.056			D															
103	H704_2065_060_RE_p002s	B	0.171	0.037																		N
104	H704_2839_140_RE_cs	B	0.173	0.016			D				F											N
105	CNE20	BC	0.182	0.039												E						
106	H703_1026_120Es_A5	C	0.182	0.047								T										
107	HIV-0013095-2.11	C	0.189	0.054			D									V						
108	H703_1687_100Es	C	0.189	0.084			D		V					L								N
109	H703_0109_210s	C	0.193	0.087			D				F											N
110	0260.v5.c36	A	0.195	0.175								A										
111	H704_0726_080sN	B	0.196	0.055								P										
112	C2101.c01	AE	0.198	0.064																		N
113	TRJO4551.58	B	0.200	0.044			D							R								N
114	C4118.c09	AE	0.202	0.051																		N
115	H704_2981_150_RE_pblib005_s	B	0.202	0.061										K								N
116	H704_1991_230_RE_p002s_1194T	B	0.203	0.039										R								
117	6952.v1.c20	CD	0.204	0.111			D		T	N		P										N
118	V703_2141_160_RE_sga3D6_s	C	0.206	0.130			D		T								W					N
119	T251-18	AG	0.206	0.068																		
120	H704_2834_210_RE_pb001_s	B	0.215	0.047										R								
121	H704_1429_090_eN1A	B	0.217	0.009			D															
122	R3265.c06	AE	0.219	0.147																		N
123	X2131_C1_B5	G	0.222	0.242			D									V						
124	H704_0746_760_RE_p002s	B	0.233	0.054				S	V													
125	246F_C1G	C	0.234	0.313			D		V		F											N
126	H704_1991_230_RE_p001s_1194T	B	0.245	0.052										R								
127	62357_14_D3_4589	B	0.248	0.064																		
128	H703_0860_150Es	C	0.252	0.008							F			L		V						
129	H704_0746_760_RE_p001s	B	0.268	0.058				S	V													
130	H704_0746_760_RE_p003s	B	0.270	0.066				S	V													
131	H704_0513_150_RE_pblib003_s	B	0.271	0.064			D		T		F	A										
132	Ce2010_F5	C	0.278	0.057																		
133	ZM214M.PL15	C	0.283	0.182																		
134	H704_2834_210_RE_pb002_s	B	0.293	0.065										R								
135	H703_1714_080c	C	0.305	0.006			D		T			P				E						
136	6244_13_B5_4576	B	0.310	0.182										L								
137	SC05_8C11_2344	B	0.321	0.127			D					A										
138	BJOX010000.06.2	AE	0.323	0.532			D															N
139	V703_0309_100_RE_pblib002_s	C	0.328	0.017					V													N

bioRxiv preprint doi: https://doi.org/10.1101/2025.10.22.684031; this version posted October 23, 2025. The copyright holder for this preprint (which was not certified by peer review) is the author/funder, who has granted bioRxiv a license to display the preprint in perpetuity. It is made available under aCC-BY 4.0 International license.

Supplementary Table 11. (continued)

#	HIV strain	Clade	IC_{80}		Amino acid in the HIV Env gp120 (BG505) position that contacts N49P7-FR (□) and/or eN49P7-FRv1-23 VH (■)																	
			N49 P7-FR	eN49 P7-FR	P118 ■	K207 □	N279 □■	N280 □	A281 ■	K282 □■	Y318 □	S365 □■	D368 □■	M426 ■	Q428 ■	T455 ■	R456 □■	D457 □■	G458 □■	P470 ■	G472 □	D474 ■
140	H704_1429_090_eN2G	B	0.329	0.012			D															
141	QH0692.42	B	0.342	0.287																		
142	V703_1855_162_RE_pblib003_s	C	0.342	0.020					V		F											
143	P1981_C5_3	G	0.360	0.376			D									V						
144	BJOX009000.02.4	AE	0.364	0.573			D		T													N
145	1056_10_TA11_1826	B	0.371	0.100			D															N
146	H704_0128_220_RE_pb001_s	B	0.405	0.015			D															N
147	CNE17	BC	0.410	0.129			D									V						
148	H704_0886_250_RE_p001s	B	0.433	0.076			D															
149	Ce703010054_2A2	C	0.437	0.203			D		V							V						N
150	7030102001E5(Rev-)	C	0.438	0.005			D															N
151	C1080.c03	AE	0.452	0.197																		N
152	H704_0026_231_RE_pbsga001_s	B	0.454	0.024			D		T													
153	H704_3000_240_RE_pbsga001_s	B	0.482	0.021				T	V		F											
154	H704_0513_150_eN01T	B	0.487	0.074			D		T		F	A										
155	T257-31	AG	0.492	0.243																		
156	H703_0309_100s	C	0.507	0.025					V													N
157	Ce1086_B2	C	0.526	0.123												L						
158	X1254_c3	G	0.616	0.273			D		T		F					V						
159	CNE30	BC	0.625	0.132							F					V						
160	Du422.1	C	0.648	0.058					I								W					N
161	Du172.17	C	0.657	0.049							F											
162	H704_2981_150_RE_pblib003_s	B	0.661	0.068										K								N
163	H704_0575_060_EsN2T	B	0.689	0.096			D		T		F											N
164	A07412M1.vrc12	D	0.728	0.142																		
165	H704_0575_060_EsN1A	B	0.740	0.091			D		T		F											N
166	H704_0575_060_RE_p002s	B	0.770	0.050			D		T		F											N
167	Ce2060_G9	C	0.792	0.309																		
168	ZM233M.PB6	C	0.816	>50			D		V													
169	Ce704809221_1B3	C	0.852	0.179																		
170	6240_08_TA5_4622	B	0.903	0.187																		
171	H704_3000_240_RE_pbsga002_s	B	0.940	0.022				T	V		F											
172	CNE5	AE	0.962	0.190			D															N
173	Ce1176_A3	C	0.980	0.140																		
174	ZM197M.PB7	C	1.069	0.071			D		T		F	A										N
175	H704_2981_150_RE_p002s_2559A	B	1.180	0.175										K								N
176	6405.v4.c34	D	1.344	0.202			D		T		W											N
177	6535.3	B	1.662	0.312			D							R		A						
178	HIV-16845-2.22	C	2.013	0.549												V						
179	231965.c01	D	2.187	0.182			D		T		F											
180	ZM53M.PB12	C	2.334	0.273							F											
181	H704_2767_070sN	B	2.711	>50			E							R								N
182	H704_0911_150sN	B	3.177	0.321			D															
183	CNE21	BC	3.270	0.043					V							E						
184	H704_2981_150_RE_p001s_2559A	B	3.304	0.590										K								N
185	6540.v4.c1	AC	4.031	4.480				S									S		Y			
186	CAAN5342.A2	B	4.198	0.719			D									V						
187	1054_07_TC4_1499	B	4.223	0.927			D															
188	ZM135M.PL10a	C	6.379	0.157			D		T													
189	CAP45.2.00.G3	C	6.962	0.057					I													N
190	T250-4	AG	7.483	0.097			D															
191	H704_1109_140_RE_cs	B	8.196	0.622			D				F											N
192	191955_A11	A	9.224	0.024							F											
193	3817.v2.c59	CD	12.494	3.744																		
194	AC10.0.29	B	16.531	0.761						R												
195	CAP210.2.00.E8	C	34.836	0.913					V			V										
196	THRO4156.18	B	38.453	2.846							F	A										
197	6041.v3.c23	AC	>50	0.019																		
198	6480.v4.c25	CD	>50	0.041					V													
199	H704_0445_180_RE_con_s	B	>50	0.098			E		T			P					K					N
200	Ce1172_H1	C	>50	0.127																		
201	6811.v7.c18	CD	>50	0.247					V													
202	1394C9G1(Rev-)	C	>50	0.359																		
203	3103.v3.c10	ACD	>50	4.713			D		V													N
204	X1632_S2_B10	G	>50	38.264			D															
205	T278-50	AG	>50	>50			A															N
206	620345.c01	AE	>50	>50			K		T								S					
